# Effectiveness of a training program for police officers who come into contact with people with mental health problems: A pragmatic randomised controlled trial

**DOI:** 10.1371/journal.pone.0184377

**Published:** 2017-09-08

**Authors:** Arabella Scantlebury, Caroline Fairhurst, Alison Booth, Catriona McDaid, Nicola Moran, Adwoa Parker, Rebecca Payne, William J. Scott, David Torgerson, Martin Webber, Catherine Hewitt

**Affiliations:** 1 York Trials Unit, Department of Health Sciences, University of York, York, England; 2 Department of Social Policy and Social Work, University of York, York, England; 3 North Yorkshire Police, Newby Wiske Hall, Northallerton, North Yorkshire, England; TNO, NETHERLANDS

## Abstract

**Introduction:**

Police officers frequently come into contact with individuals with mental health problems. Specialist training in this area for police officers may improve how they respond to individuals with mental health problems; however, evidence to support this is sparse. This study evaluated the effectiveness of one bespoke mental health training package for frontline police officers relative to routine training.

**Design:**

Pragmatic, two-armed cluster randomised controlled trial in one police force in England. Police stations in North Yorkshire were randomised with frontline police officers receiving either a bespoke mental health training package or routine training. The primary outcome was the number of incidents which resulted in a police response reported to the North Yorkshire Police control room up to six months after delivery of training. Secondary outcomes included: likelihood of incidents using Section 136 of the Mental Health Act; likelihood of incidents having a mental health tag applied; and number of individuals with a mental health warning marker involved in incidents. The appropriateness of mental health tags applied to a random sample of incidents was checked by an independent mental health professional. Routinely collected data were used.

**Results:**

Twelve police stations were recruited and randomised (Intervention group n = 6; Control group n = 6), and 249 officers received the bespoke mental health training intervention. At follow-up, a median of 397 incidents were assigned to trial stations in the intervention group, and 498 in the control group. There was no evidence of a difference in the number of incidents with a police response (adjusted incidence rate ratio (IRR) 0.92, 95% CI 0.61 to 1.38, p = 0.69), or in the number of people with mental health warning markers involved in incidents (adjusted IRR 1.39, 95% CI 0.91 to 2.10, p = 0.13) between the intervention and control groups up to six months following the intervention; however, incidents assigned to stations in the intervention group were more likely to have a mental health tag applied to them than incidents assigned to control stations (adjusted odds ratio 1.41, 95% CI 1.16 to 1.71, p = 0.001). The review of 100 incidents suggests that there may be incidents involving individuals with mental health issues that are not being recorded as such (Kappa coefficient 0.65). There was no statistically significant difference in the likelihood of Section 136 of the Mental Health Act being applied to an incident.

**Conclusions:**

The bespoke one day mental health training delivered to frontline officers by mental health professionals did not reduce the number of incidents reported to the police control room up to six months after its delivery; however training may have a positive effect on how the police record incidents involving individuals with mental health problems. Our trial has shown that conducting pragmatic trials within the police setting is feasible and acceptable. There is a wealth of routinely collected police data that can be utilised for research and further collaboration between police forces and academia is encouraged.

**Trial registration:**

ISRCTN (ISRCTN11685602). The authors confirm that all ongoing and related trials for this drug/intervention are registered.

## Introduction

Mental illness constitutes an estimated 7.4% of the world’s measurable burden of disease [1], with the economic impacts associated with mental disorders greater than those related to each of the four other major categories of non-communicable diseases: diabetes, cardiovascular diseases, chronic respiratory diseases, and cancer [2].

Police officers are often the first to respond to incidents involving individuals with mental health problems in crisis [3]. Although national data are not available, regional police forces routinely record mental health issues through mental health ‘warning markers’ which are applied to an individual’s record to indicate that they have mental health problems, and mental health tags, which indicate that mental health is a factor in an incident. These data have been used by the College of Policing (CoP) to estimate that approximately 15–20% of police time is spent on incidents linked to mental health in England and Wales [4]. At a time when there have been significant cuts to mental health services, the amount of police time spent responding to incidents involving individuals with mental health problems has led to concerns that police officers are being relied on as a ‘first resort’, which is not only placing strain on the police but leading to concerns for public safety [5] [6]. As a result, the UK government have pledged to invest an additional £1billion in mental health services by 2020 [6] and the UK police service is introducing a number of initiatives to help police officers deal with the rising number of incidents involving individuals with mental ill health. For example, Street Triage has been piloted in a number of police forces throughout England and aims to improve how police officers respond to people with possible mental health problems through collaboration with mental health professionals [7].

Political interest in how the police record and respond to incidents involving individuals with mental health has increased following a recent report into the police’s use of Section 136 of the Mental Health Act (MHA), which gives officers the power to remove anyone who appears to be suffering from mental health problems from a public place to a place of safety (PoS) (e.g., a hospital, or police custody when there is no health-based PoS (HBPoS) available) [8]. The report concluded that, contrary to guidance, in some areas police custody is being regularly used as a PoS. This was mostly attributed to: insufficient staff and available beds at a HBPoS; the person having consumed alcohol; or displaying and/or having a history of violence. However, the report also identified gaps in knowledge and variations in the amount of training police officers had received around Section 136 of the MHA [8].

Mental health training may improve how police officers respond to and record situations involving individuals with mental health problems [9]. A recent systematic review of the effectiveness of mental health training programmes for non-mental health trained professionals, including the police, reported that there were huge variations in training design, delivery and content, making the best approaches to training unclear. The review also stated that due to the poor quality of existing evidence, it is difficult to draw conclusions on the effectiveness of mental health training for police, with the review identifying only short term changes in behaviour [10]. However, there was some evidence to suggest that training by mental health professionals could be beneficial. Despite the uncertain evidence base surrounding mental health training and policing, training interventions are being introduced into police forces worldwide. For example, Training and Education about Mental Illness for Police Organisations (TEMPO) [11] is being introduced into police forces in Canada and the Crisis Intervention Team (CIT) programme [12] is being introduced into police forces in the USA and elsewhere. In the UK, police forces are required to adhere to the CoP standards for mental health training provision, but have autonomy to decide how training is delivered and so the amount of training received varies across forces [9].

In this paper we report on the findings from a randomised controlled trial (RCT) of the effectiveness of a face-to-face mental health training intervention delivered by mental health practitioners to frontline police officers in addition to routine training, compared to routine training only in reducing demand on police time.

## Methods

### Study design

We conducted a pragmatic, two-armed cluster RCT to assess the effectiveness of a specialised mental health training intervention relative to routine training for frontline officers, with North Yorkshire Police (NYP) stations as the clusters, and a six month follow-up. A cluster randomised design was chosen to minimise contamination between police officers as there is much less interaction between officers at different stations than within stations. Individually randomising police officers was not considered appropriate as those in the control arm may have been partially exposed to the intervention through interaction with officers receiving the intervention. Additionally, police officers often work in pairs or groups and so individually randomising officers posed a particular contamination risk if intervention officers discussed the training with their partners/team members allocated to the control group. The study was approved by the North Yorkshire Police Training Commissioning Group on 14^th^ January 2016 and the University of York Health Sciences Research Governance Committee on 18^th^ March 2016 (HSRGC/2016/152D).

### Setting

Within the NYP force there are 39 police stations operating within six ‘Safer Neighbourhood Command’ areas (SNCs). Within each SNC, the two police stations with the highest number of frontline police officers were randomised for eligible frontline officers reporting to that station to receive either the specialised mental health training package (intervention) or routine training only (control). The reasons for selecting the two largest stations within each of the six SNCs were twofold. First, it was not considered feasible for all 39 police stations to take part since it would have been difficult to deliver the specialised training to half of these stations within the project time frame. Second, smaller police stations have fewer staff and irregular opening hours, to accommodate this there is greater movement of officers between smaller stations than the larger stations. For instance, the small number of staff operating within smaller stations means that if officers are not available (e.g. through sickness) officers from larger stations are sent to cover the deficit; smaller stations were therefore considered to pose a more significant contamination risk.

### Eligibility and recruitment

In February 2016 the NYP training department assisted the research team in recruiting 12 police stations (the two with the largest number of frontline officers within each SNC). The Mental Health Partnership Development Inspector for NYP identified eligible police stations by manually extracting details of the rank and number of frontline officers within each police station from NYP’s IT system. The 12 stations were subsequently randomised and the NYP training department informed each station allocated to the intervention group about the training. Training for police officers is mandatory and so following approval from the police force’s training commissioning group, participation in the training intervention was made compulsory for eligible frontline officers reporting to stations that were randomised to the intervention group. NYP’s Resource Management Unit (RMU) extracted officers from duty to attend the training (with a minimum of three months’ notice). Frontline police officers were eligible for trial participation if they were Response or Safer Neighbourhood Officers within the ranks: Police Constable (PC), Sergeant and Inspector. Police Community Support Officers (PCSOs) were also eligible for the trial. Definitions of the roles and responsibilities of included officers are provided in S1 File (e.g. fire-arms and dog handlers) as these individuals often move between police stations. Call handlers, control room and custody staff were also excluded as the training was aimed at frontline officers. Officers reporting to stations randomised to the control group received routine training only. All police stations and officers recruited to the trial received an information sheet.

### Randomisation

Randomisation was undertaken by a statistician at the York Trials Unit (YTU).

Allocation occurred at the cluster level with police stations randomised 1:1 to either the intervention or control group. Minimisation was used via a dedicated computer programme, MinimPy [13], to ensure that the groups were balanced in terms of: number of frontline officers, including specialist roles (dichotomised as <43, ≥43 officers, which was the median of the batch), SNC (Scarborough, York, Harrogate, Hambleton and Richmondshire, Selby, and Craven) and whether Street Triage was operational in that area (Yes/No). Street Triage is in operation within three of the six SNC and involves close collaboration between police and mental health services and so may affect how frontline officers respond to incidents involving mental health in these areas. Following randomisation, the NYP training department informed eligible officers at stations randomised to the intervention group about the study and arranged for them to attend training.

### Control

All NYP officers received training on mental health between January and March 2016. This training was an adapted version of training that was produced by Thames Valley Police and Oxfordshire Mind. The training covered basic mental health law, NYP procedures around mental health and responding to incidents involving individuals with mental health problems. All officers must also undertake a 2–3 hour online mental health training package as part of their basic training at the start of their career. Officers allocated to the control group were not informed of their allocation and did not receive any additional training outside of this mandatory mental health training.

### Intervention

In addition to routine mental health training, eligible frontline officers were extracted from duty to attend a face-to-face one-day bespoke mental health training programme delivered in a classroom setting by qualified and experienced mental health professionals from the local NHS mental health trust. The intervention was delivered across 25 training days at 3 police locations in North Yorkshire between May and August 2016. The training aimed to enhance officers’ understanding of and ability to: identify mental vulnerability; record relevant information using available systems; respond using appropriate internal and external resources; refer vulnerable people into services to provide longer-term assistance; and review incidents to make sure that risks have been effectively managed. The timing of the intervention’s delivery was decided with the NYP training department to ensure that there was sufficient time for the training department to organise the release of officers to attend the training and that the training was delivered within the project’s time frame to allow for the six month follow-up period. The intervention was developed by researchers at the University of York in conjunction with mental health practitioners from the local NHS mental health trust and NYP. The content of the training was informed by the College of Policing (CoP) Learning Standards, which provide a framework for mental health training for police officers, and a systematic review of mental health training for non-mental health trained professionals [10]. Findings from the systematic review suggest that training is most effective when it is delivered by mental health professionals using a variety of different delivery methods [10]. The mental health training intervention was therefore delivered to frontline officers by mental health professionals using: lecture style delivery; small group discussion; filmed scenarios; short films with experts that had experience of living with a mental health condition and contact with NYP during a mental health crisis; and talking head videos with 11 mental health services and partner agencies. A summary of the intervention details is provided in S1 Table.

### Blinding

Due to the nature of the intervention, it was not feasible to blind police stations and individual participants to the group they were allocated to; however, stations allocated to the control group were not explicitly informed of their allocation.

### Outcomes

The pre-specified primary outcome was the number of incidents reported to the NYP control room which resulted in a police response. Secondary outcomes included: likelihood of incidents having Section 136 of the MHA applied; likelihood of incidents having a mental health tag applied; and number of individuals with a mental health warning marker involved in any incident.

Routinely collected police call data were used to assess these outcomes. All calls made to NYP were automatically recorded on the in-house IT system (STORM) and are then transferred to the Niche system. Data were extracted from the Niche and STORM systems using ibase (IMB) by an NYP intelligence analyst. Calls about the same incident are linked, as far as is possible, by a unique incident identifier. Incident-level data on all incidents reported to NYP in a four-week period were collected from the Niche system before (April 2016) and approximately six months after (mid-November to mid-December, to avoid the Christmas period) the delivery of the training. The data included: unique incident number, incident type, date and time reported, a unique identifier for the individual reporting the incident where available, number of individuals involved, the number of the individuals involved who have a mental health warning marker applied to them by call handlers during calls or following requests from officers, whether the call handler applied a mental health tag to the incident, and whether Section 136 of the MHA was applied (split by whether the individual(s) was held in custody or taken to a HBPoS). For each incident, details of the officer(s) who attended the incident were received including their rank and the station they report to. The assigned “Officer in Case” (‘OIC’–the lead officer in the incident) was indicated for each incident where available.

A random sample of 100 incidents (50 from the baseline sample and 50 from follow-up) were reviewed by an independent mental health professional to assess whether or not a mental health tag should have been applied to the incident. This review was conducted blind to whether or not a tag was actually applied. When conducting the review, the mental health professional was provided with the same information that force control room staff used to decide whether an incident should have a mental health tag applied. This included: the incident occurrence number and the ‘CAD log’. The CAD log is a transcript that is recorded during live incidents (e.g. calls) by force control room call handlers. The transcript includes a record of the callers’ report, the force control room call handlers’ comments and actions, and officer incident reports. Officers may update CAD logs with incident information via the force control room or can add further details to the NICHE record, once it has been converted from STORM at the closure of the ‘at-scene’ incident.

NYP officers’ knowledge, attitudes, understanding, confidence and response to individuals with mental health problems were also evaluated through a survey and qualitative interviews, the results of which will be reported elsewhere (manuscript submitted).

### Sample size

No formal power calculation was conducted for this trial. We recruited and randomised 12 NYP stations. At least 4 clusters per arm are recommended for a cluster RCT[14], and our sample size exceeds this minimum recommendation (with six clusters per arm) whilst allowing for resource constraints (i.e., considering the number of frontline officers it was feasible to train during the intervention delivery period).

### Statistical analysis

Analyses were conducted in Stata version 13[15], using two-sided statistical tests at the 5% significance level. Stations were analysed in the groups to which they were originally allocated, irrespective of whether the individual officers received the training or not, under the principles of intention to treat.

The minimisation factors of the randomised stations are summarised descriptively overall and by trial arm. No formal statistical comparisons were undertaken[16, 17]. Data on attendance at the training events are described.

All incidents reported to NYP between 1^st^ and 30^th^ April 2016, and between 15^th^ November and 14^th^ December 2016 were extracted, and all incidents attended by at least one officer from a station participating in the trial were retained for analysis. Each incident was assigned to a trial station based on the station that the OIC reported to. Where an incident did not have an OIC or the OIC was from a non-trial station, the incident was assigned to the trial station the attending officers most commonly reported to. Post-hoc sensitivity analyses, suggested by the reviewer, were undertaken for the primary and secondary outcomes whereby incidents were assigned to a trial station based purely on the trial station the attending officers most commonly reported to.

#### Primary analysis

The primary analysis compared the number of incidents with a police response per station between the intervention and control groups using negative binomial regression at the station level adjusting for number of incidents with a response at baseline, and the minimisation factors of number of frontline officers (as a continuous variable), and SNC. It was not necessary to include Street Triage in the models as this was nested within SNC, that is, each of the two stations within each SNC were either both implementing Street Triage or neither were. The adjusted incidence rate ratio (IRR) is presented with a 95% confidence interval (CI) and p value, where an IRR of less than 1 indicates a decrease in the predicted number of recorded incidents that require a police response in stations allocated to the intervention group relative to those allocated to the control group.

The likelihood of an incident having a mental health tag applied was analysed at the incident level using a mixed logistic regression model adjusting for the police station level covariates of proportion of incidents with a mental health tag applied at baseline, number of frontline officers, and SNC as fixed effects, and for police station as a random effect. An analogous approach was taken for the likelihood of an incident having Section 136 of the MHA applied (either in custody or a HBPoS). Since the number of incidents with Section 136 applied was low, a post hoc sensitivity analysis was conducted using penalised logistic regression for rare events (model adjusted as described except police station included as a fixed effect as random effects not permitted). For each of these analyses, the odds ratio (OR) for the intervention effect is presented with a 95% CI and p-value, where an OR of greater than 1 indicates an increased likelihood of the event in the intervention group relative to the control group.

#### Secondary analysis

The total number of individuals with a mental health warning marker involved in any incident with a police response was analysed at the station level in a similar way to the primary outcome, with an additional covariate for the average number of officers in attendance at an incident. The adjusted IRR is presented with a 95% CI and p-value, and is interpreted in an analogous way to that described for the primary analysis.

The agreement between the application of mental health tags to incidents by the original assignment and the mental health professional was assessed using Cohen’s kappa coefficient [18].

### Protocol changes

The study protocol was registered in June 2016 (ISRCTN11685602) and the protocol was made available on our project website [19] (S2 File). In the protocol, the primary outcome was defined at *“the person-level*, *where each person is a member of the public who makes at least one call to the control room which results in a police response (known as a caller)*.*”*

However, it became clear that calls about the same incident are linked together by a unique incident identifier so the data we received was at the incident-level rather than the caller-level. We aggregated the number of incidents reported to the station-level for analysis. The change in the unit of analysis from caller-level to incident-level was necessary as many incidents did not have a caller assigned to them, for instance not all callers provide identifying details.

It was originally planned to consider the number of “frequent” callers as a secondary outcome; however, data for this could not be obtained due to issues with how the data are recorded and not being able to extract data in a format that was suitable for analysis.

In the protocol we stated that, data permitting, the analysis of mental health tags would be additionally adjusted for attending officer characteristics (such as average years in service, male/female/mixed, average age); however, it was not possible to obtain this level of detail about the attending officers.

## Results

**Fig 1:**
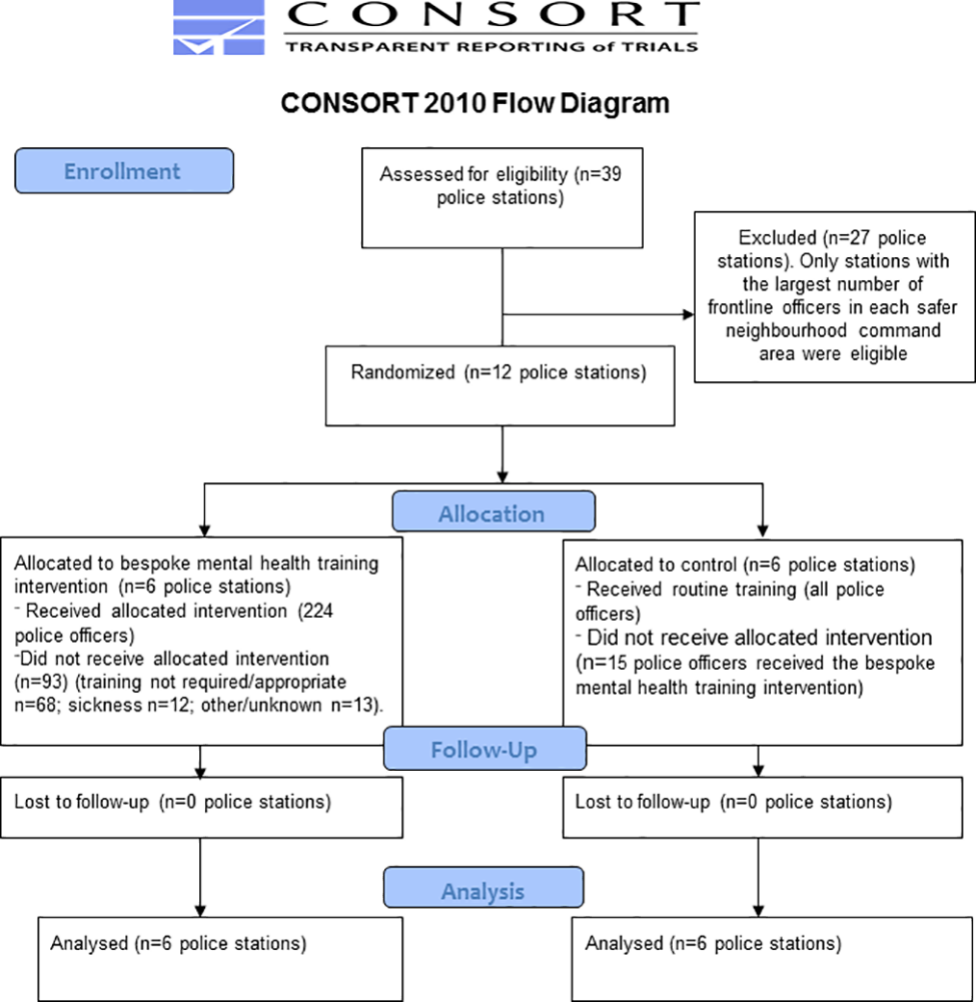
CONSORT flow diagram.

Twelve police stations across North Yorkshire were recruited and randomised into the trial; six to the intervention group and six to the control group (Fig 1). The median number of frontline officers at these stations was 43 (range 10 to 147). The intervention and control groups were balanced on number of frontline officers, SNC and Street Triage (Table 1). The total number of officers put forward for training was 360. Of these, 249 (69.1%) officers received the specialised mental health training intervention at one of 25 training events; 224 from stations allocated to the intervention group, 15 from stations allocated to the control group, and 10 from non-trial stations. Each training day was attended by a median of 9 officers (range 3 to 20). Reasons for non-attendance included: training not required/appropriate n = 81 (ineligible rank/role n = 71; left force n = 5; officer on maternity adjustment n = 3, restricted duties n = 1, or due to retire n = 1); sickness n = 13; and other/unknown n = 17. Nineteen attendees were trained inappropriately as they were not of a rank eligible for the intervention (intervention group n = 15; control group n = 2; non-trial n = 2). Therefore, 230 eligible frontline officers were trained (intervention group n = 209; control group n = 13; non-trial n = 8). These included: constables (n = 148, 64.4%); PCSOs (n = 51, 22.2%); and sergeants (n = 28, 12.2%). The rank/role of one attendee was missing.

**Table 1:** Minimisation factors of the participating police stations.

Characteristics	Intervention(n = 6)	Control(n = 6)	Total(n = 12)
**No. of frontline officers, n (%)**			
<43	3 (50.0)	3 (50.0)	6 (50.0)
>43	3 (50.0)	3 (50.0)	6 (50.0)
Mean (SD)	54.7 (50.3)	65.7 (54.1)	60.2 (50.1)
Median (min, max)	45.5 (12, 147)	42.5 (10, 141)	42.5 (10, 147)
**Safer Neighbourhood Command area, n (%)**			
Craven	1 (16.7)	1 (16.7)	2 (16.7)
Hambleton and Richmondshire	1 (16.7)	1 (16.7)	2 (16.7)
Harrogate	1 (16.7)	1 (16.7)	2 (16.7)
Scarborough	1 (16.7)	1 (16.7)	2 (16.7)
Selby	1 (16.7)	1 (16.7)	2 (16.7)
York	1 (16.7)	1 (16.7)	2 (16.7)
**Street Triage, n (%)**			
Yes	3 (50.0)	3 (50.0)	6 (50.0)
No	3 (50.0)	3 (50.0)	6 (50.0)

SD, standard deviation

Data were received on 9157 incidents reported to NYP in April 2016 that required a police response, of these 6665 (72.8%) were attended by at least one officer from a police station involved in the trial: 3208 (48.1%) of these incidents were assigned to a station allocated to the intervention group; and 3457 (51.9%) to a station allocated to the control group. Between 189 and 264 eligible incidents were reported on each day of the month, and more incidents were reported on a Saturday than any other day of the week (Table 2). Most incidents in each group were attended to only by officers reporting to stations allocated to that group (n = 2033, 63.4% in the intervention group; n = 2013, 58.2% in the control group). The most commonly reported types of incident were public safety and welfare (PSW) concerns (n = 861, 12.9%). One in 10 incidents had a mental health tag applied to them (n = 655, 9.8%) and Section 136 of the MHA was applied in 17 cases (0.3%). For most of these, the individual(s) were taken to a HBPoS (n = 15) rather than being retained in custody. The incidents assigned to the intervention and control groups at baseline appear broadly comparable, except that there was a higher number of Section 136s in the control group (n = 13, 0.4% vs n = 4, 0.1%).

**Table 2:** Characteristics of incidents attended by at least one officer from a station participating in the trial over one month prior to the delivery of a mental health training programme by trial arm.

Baseline incidents	Intervention(n = 3208)	Control(n = 3457)	Total(n = 6665)
**No. per day**			
Mean (SD)	106.9 (13.8)	115.2 (12.3)	222.2 (21.7)
Median (min, max)	107.5 (85, 135)	114.5 (87, 136)	220.5 (189, 264)
**Day of the week, n (%)**			
Monday	421 (13.1)	424 (12.3)	845 (12.7)
Tuesday	376 (11.7)	446 (12.9)	822 (12.3)
Wednesday	387 (12.1)	434 (12.6)	821 (12.3)
Thursday	429 (13.4)	433 (12.5)	862 12.9)
Friday	558 (17.4)	597 (17.3)	1155 (17.3)
Saturday	615 (19.2)	646 (18.7)	1261 (18.9)
Sunday	422 (13.2)	477 (13.8)	899 (13.5)
**Incident type, n (%)**			
PSW Concern for Safety/Collapse/Injury/Illness/Trapped	426 (13.3)	435 (12.6)	861 (12.9)
PSW Suspicious Circumstances/Insecure Premises/Vehicle	401 (12.5)	454 (13.1)	855 (12.8)
ASB Nuisance	407 (12.7)	396 (11.5)	803 (12.1)
Crime Violence	221 (6.9)	246 (7.1)	467 (7.0)
Admin Police Generated Resource Activity	167 (5.2)	192 (5.6)	359 (5.4)
Other	1586 (49.4)	1734 (50.2)	3320 (49.8)
**Attended by officers reporting to, n (%)**	0 (0.0)	2013 (58.2)	2013 (30.2)
Control stations only	2033 (63.4)	0 (0.0)	2033 (30.5)
Intervention stations only	0 (0.0)	1,018 (29.5)	1018 (15.3)
Control and non-trial stations	815 (25.4)	0 (0.0)	815 (12.2)
Intervention and non-trial stations	219 (6.8)	190 (5.5)	409 (6.1)
Intervention and control stations			
Intervention, control and non-trial stations	141 (4.4)	236 (6.8)	377 (5.7)
**Rank/role of OIC, n (%)**			
Detective Inspector/Inspector	8 (0.3)	9 (0.3)	17 (0.3)
Detective Sergeant/Sergeant	101 (3.2)	167 (4.8)	268 (4.0)
Detective Constable/Constable	2384 (74.3)	2513 (72.7)	4897 (73.5)
Special Inspector/Special Sergeant/Special Constable	25 (0.8)	53 (1.5)	78 (1.2)
No OIC assigned	690 (21.5)	715 (20.7)	1405 (21.1)
**No. of individuals involved, n (%)**			
0	635 (19.8)	692 (20.0)	1327 (19.9)
1	1202 (37.5)	1279 (37.0)	2481 (37.2)
2–4	1202 (37.5)	1314 (38.0)	2516 (37.8)
5–9	157 (4.9)	161 (4.7)	318 (4.8)
10+	12 (0.4)	11 (0.3)	23 (0.4)
Median (min, max)	1 (0, 36)	1 (0, 22)	1 (0, 36)
**No. of individuals involved with mental health warning marker**			
0	2604 (81.2)	2873 (83.1)	5477 (82.2)
1	520 (16.2)	487 (14.1)	1007 (15.1)
2–4	83 (2.6)	97 (2.8)	180 (2.7)
5–9	1 (0.03)	0 (0.0)	1 (0.02)
Median (min, max)	0 (0,5)	0 (0,4)	0 (0, 5)
**Mental health tag, n (%)**	317 (9.9)	338 (9.8)	655 (9.8)
**Section 136 MHA applied, n (%)**			
Custody	1 (0.03)	1 (0.03)	2 (0.03)
HBPoS	3 (0.1)	12 (0.4)	15 (0.2)
Total	4 (0.1)	13 (0.4)	17 (0.3)

A total of 8434 incidents were reported to NYP between 15^th^ November and 14^th^ December 2016 (inclusive), of which 6353 (75.3%) were attended by at least one officer from a police station involved in the trial; 2860 (45.0%) in the intervention group; and 3493 (55.0%) in the control group (Table 3). As at baseline, most incidents in each group were attended to only by officers reporting to stations allocated to that group (n = 1796, 62.8% in the intervention group; n = 2228, 63.8% in the control group).

**Table 3:** Characteristics of incidents attended by at least one officer from a station participating in the trial over one month, six months following the start of the delivery of a mental health training programme, by trial arm.

Follow-up incidents	Intervention(n = 2860)	Control(n = 3493)	Total(n = 6353)
**No. per day**			
Mean (SD)	95.4 (13.6)	117.5 (13.3)	211.8 (23.5)
Median (min, max)	95 (71, 138)	117 (94, 146)	207.5 (167, 270)
**Day of the week, n (%)**			
Monday	372 (13.0)	409 (11.7)	781 (12.3)
Tuesday	440 (15.4)	551 (15.8)	991 (15.6)
Wednesday	420 (14.7)	557 (16.0)	977 (15.4)
Thursday	387 (13.5)	431 (12.3)	818 (12.9)
Friday	375 (13.1)	496 (14.2)	871 (13.7)
Saturday	472 (16.5)	513 (14.7)	985 (15.5)
Sunday	394 (13.8)	536 (15.3)	930 (14.6)
**Incident type, n (%)**			
PSW Concern for Safety/Collapse/Injury/ Illness/Trapped	436 (15.2)	490 (14.0)	926 (14.6)
PSW Suspicious Circumstances/Insecure Premises/Vehicle	366 (12.8)	410 (11.7)	776 (12.2)
ASB Nuisance	286 (10.0)	347 (9.9)	633 (10.0)
Crime Violence	215 (7.5)	251 (7.2)	466 (7.3)
Road Related Offence	181 (6.3)	192 (5.5)	367 (5.8)
Other	1376 (48.1)	1803 (51.6)	3185 (50.1)
**Attended by officers reporting to, n (%)**			
Control stations only	0 (0.0)	2228 (63.8)	2228 (35.1)
Intervention stations only	1796 (62.8)	0 (0.0)	1796 (28.3)
Control and non-trial stations	0 (0.0)	82 (23.6)	824 (13.0)
Intervention and non-trial stations	684 (23.9)	0 (0.0)	684 (10.8)
Intervention and control stations	228 (8.0)	264 (7.6)	492 (7.7)
Intervention, control and non-trial stations	152 (5.3)	177 (5.1)	329 (5.2)
**Rank/role of OIC, n (%)**			
Chief Inspector	0 (0.0)	1 (0.03)	1 (0.02)
Detective Inspector/Inspector	2 (0.1)	4 (0.1)	6 (0.1)
Detective Sergeant/Sergeant	97 (3.4)	134 (3.8)	231 (3.6)
Detective Constable/Constable	2032 (71.1)	2388 (6834)	4420 (69.6)
Student Constable	157 (5.5)	252 (7.2)	409 (6.4)
PCSO	245 (8.6)	258 (7.4)	503 (7.9)
Special Inspector/Special Sergeant/Special Constable	17 (0.6)	59 (1.7)	76 (1.2)
No OIC assigned	310 (10.8)	397 (11.4)	707 (11.1)
**No. of individuals involved, n (%)**			
0	539 (18.9)	715 (20.5)	1254 (19.7)
1	1019 (35.6)	1181 (33.8)	2200 (34.6)
2–4	1153 (40.3)	1438 (41.2)	2591 (40.8)
5–9	138 (4.8)	156 (4.5)	294 (4.6)
10+	11 (0.4)	3 (0.1)	14 (0.2)
Median (min, max)	1 (0,14)	1 (0,12)	1 (0, 14)
**No. of individuals involved with mental health warning marker**			
0	2321 (81.2)	2951 (84.5)	5272 (83.0)
1	459 (16.1)	468 (13.4)	927 (14.6)
2–4	80 (2.8)	74 (2.1)	154 (2.4)
Median (min, max)	0 (0, 3)	0 (0, 4)	0 (0, 4)
**Mental health tag applied, n (%)**	349 (12.2)	326 (9.3)	675 (10.6)
**Section 136 MHA applied, n (%)**			
Custody	2 (0.1)	1 (0.03)	3 (0.05)
HBPoS	11 (0.4)	13 (0.4)	24 (0.4)
Total	13 (0.5)	14 (0.4)	27 (0.4)

Incident type: five most common incident types (overall) listed

SD, standard deviation; PSW, Public Safety and Welfare; ABS, Anti-social behaviour; PCSO, Police Community Support Officer; OIC, Officer in Case; MHA, Mental Health Act; HBPoS, Health Based Place of Safety

A median of 373 incidents were assigned to trial stations in the intervention group at baseline, and 397 at follow-up (Table 4). The corresponding figures for the control group are 431 and 498, respectively. There was no evidence of a difference in the number of incidents with a police response between the intervention and control groups following the intervention (adjusted IRR 0.92, 95% CI 0.61 to 1.38, p = 0.69).

**Table 4:** Summary of police station-level outcomes.

Station-level outcomes	Baseline		Follow-up	
	Intervention(n = 6)	Control(n = 6)	Intervention(n = 6)	Control(n = 6)
**No. of incidents**				
Mean (SD)	534.7 (476.9)	576.2 (436.3)	476.7 (376.9)	582.2 (409.4)
Median (min, max)	372.5 (136, 1422)	430.5 (67, 1209)	397 (133, 1168)	497.5 (54, 1144)
**No. of individuals with a mental health warning marker applied to an incident**				
Mean (SD)	118.2 (154.4)	117.3 (112.3)	105.2 (124.2)	105.5 (108.5)
Median (min, max)	55 (12, 415)	64.5 (3, 271)	56.5 (16, 341)	62 (5, 285)

SD’ standard deviation

At follow-up, 675 (10.6%) incidents (intervention group n = 349, 12.2%; control group n = 326, 9.3%) had a mental health tag applied (adjusted odds ratio (OR) 1.41, 95% CI 1.16 to 1.71, p = 0.001) and 27 (0.4%) incidents (intervention group n = 13, 0.5%; control group n = 14, 0.4%) had Section 136 applied (adjusted OR 2.75, 95% CI 0.69 to 11.02, p = 0.15; penalised logistic regression: adjusted OR 2.39, 95% CI 0.62 to 9.21, p = 0.21).

At baseline, a median of 55 individuals with a mental health warning marker were involved in incidents assigned to trial stations in the intervention group, and 65 in the control group (Table 4). At follow-up, the corresponding figures are 57 and 62, respectively (adjusted IRR 1.39, 95% CI 0.91 to 2.10, p = 0.13).

Results of the post-hoc sensitivity analyses we similar to the original analyses: number of incidents, IRR 0.93, 95% CI 0.56 to 1.45, p = 0.77; mental health tag, OR 1.29, 95% CI 1.06 to 1.58, p = 0.01; Section 136, OR 1.77, 95% CI 0.60 to 5.28, p = 0.30; number of individuals involved with a mental health tag, IRR 1.34, 95% CI 1.08 to 1.66, p = 0.01.

Of the 100 incidents randomly sampled and independently assessed, 10 had a mental health tag applied to them in practice. The independent reviewer judged that a mental health tag should have been applied to 16 of the incidents. The overall Cohen’s kappa coefficient was 0.65, indicating moderate agreement (Table 5). Where a mental health tag was applied to an incident, the blinded, independent reviewer also “applied” a mental health tag in all but one case. An additional seven cases were identified by the reviewer as ones where the application of a mental health tag was considered appropriate, but where a tag had not been applied by the call handlers.

**Table 5:** Appropriateness of application of mental health tags to random sample of 100 incidents.

*MH tag applied by independent reviewer*	Baseline(n = 50)		Follow-up(n = 50)		Total(n = 100)	
	*MH tag applied*		*MH tag applied*		*MH tag applied*	
	Yes	No	Yes	No	Yes	No
Yes	5	0	4	7	9	7
No	0	45	1	38	1	83
Cohen’s kappa	1.00		0.42		0.65	

MH, Mental Health

## Discussion

In this pragmatic cluster randomised controlled trial, we did not find that a specialised mental health training programme for frontline police officers reduced the number of incidents reported to the police control room up to six months after its delivery nor the number of individuals with a mental health warning marker involved in incidents.

There is an indication that following the delivery of the intervention incidents assigned to the intervention group were more likely to have a mental health tag applied to them than incidents assigned to control stations. This could be due to better identification of mental vulnerability by frontline officers reporting to intervention stations and therefore increased reporting and recording of such issues. The independent review of 100 incidents suggests that, where tags were used, in the majority of incidents it was appropriate; however, there may be incidents that involve individuals with mental health problems that are still not being identified. At the time of the study mental health tags could only be added to an incident by force control room staff directly, or by frontline officers requesting that a mental health tag be applied to an incident via force control room staff. There may therefore have been situations where an officer requested that a mental health tag be applied and this was not carried out.

A major complexity of analysing the routinely collected police data used in this trial was deciding how to assign incidents to a police station. Any particular incident may be attended by multiple police officers, from different police stations. We were therefore faced with the situation where incidents could be attended by officers reporting to intervention and control stations and stations not involved in the trial. It was not possible, based on the data received, to understand the extent of the role each officer played in dealing with the incident, or who had the most significant interaction with the individuals involved. The decision was made to, where possible, consider the station that the assigned OIC reported to. OICs largely tended to be police constables or PCSOs and so of a rank eligible for the specialised mental health training (however it was not possible to identify whether or not the OICs actually attended the training). If the OIC reported to a station allocated to the intervention group, then the incident was analysed in the intervention group and vice versa with the control group. Sensitivity analyses were conducted, at the request of the reviewer, whereby incidents were assigned to the trial station that officers attending the incident most commonly reported to. Results were broadly similar to the original analyses except that the point estimate for the number of individuals with a mental health tag involved in any incident was statistically significant. There are potentially many justifiable ways to define how to decide which police station to assign an incident to, and, as demonstrated, it is possible these could give differing results. Further work around this area would be informative.

There are few high quality evaluations investigating the effectiveness of specialised mental health training programmes targeted at the police [10] that we can compare our findings to. There is some evidence to suggest that training that includes dramatisations or role play and which is delivered by mental health professionals and police trainers may be beneficial [10]. A systematic review of qualitative studies found that mental health training may improve how individuals respond to situations involving mental health, their perceptions of mental health (e.g. improved empathy and reduced stigma) and their ability to recognise mental health problems (manuscript under review). Our specialised mental health training package was also evaluated through a survey and qualitative interviews with frontline officers. The findings of which suggest that the training may have improved officers’ knowledge, attitudes and confidence in responding to incidents involving individuals with mental health problems (manuscript in preparation).

### Strengths and limitations

This was a robust pragmatic cluster randomised controlled trial that evaluated a specialised mental health training package. There are relatively few trials that have been conducted within the police setting but our trial has demonstrated that it is feasible to do so. The police setting is considerably different to other contexts such as health and education and so it is recommended that when conducting research in this area that individuals within key policing roles (e.g. data analysts, senior police officers, police practitioners) are included in research teams and are involved in the design, delivery and evaluation of the research. Our trial has also highlighted that the police force routinely collect vast amounts of rich data that is relatively easy to obtain and opportunities to use these data in research may be being missed. Further collaborations between the police and academia may lead to improvements in how the police routinely collect and record data and an increase in data quality, which would in turn lead to more confidence in results produced from research using these data.

Defining appropriate outcomes for the trial was a challenge. Due to the timescales for this research, we were unable to assess the impact of the mental health training on the end-user–people with mental health problems. The police force suggested that the intervention should focus on frontline officers, with the aim of reducing the demand on police resources. There is evidence to suggest that significant strain on police resources comes from dealing with reported incidents involving people with mental ill health. If frontline officers received training in how to effectively manage such individuals, it was hoped that this would reduce the likelihood of these individuals being involved in further incidents, thereby reducing the number of incidents being reported to the police. The advantage of defining this as our primary outcome was that it could be measured using routinely collected data, and does not pose an assessment burden for the public or the police. However, the leap between the intervention and outcome is a somewhat large one and any beneficial effect could be diluted and/or take a while to observe. A longer follow-up than six months, was not possible in this study but is recommended for future studies to investigate longer-term impact. We were also required to make some changes to our pre-defined outcomes due to issues with obtaining routinely collected police data (e.g. frequent callers). The police may benefit from a review of their recording practices to ensure that the vast amounts of routinely collected data that is being collected can be utilised and is fit for purpose.

There was some contamination between the intervention and control groups as a number of officers from stations allocated to the control group inappropriately received the specialised mental health training. It is possible that this contamination could have spread beyond the actual attending officers if they discussed the training more widely with their colleagues, though quantifying this would be impossible. There was also the potential for officers to have moved station during the course of the trial but we do not have any data on the number of officers this applies to. Such contamination, as well as the fact that not all eligible officers in the intervention group received the training, could potentially have diluted any true beneficial intervention effect, should one exist. Additionally, the trial was not specifically powered to detect a particular difference in any outcome. The sample size for station-level outcomes was fixed at 12 as this was felt to be a feasible number of stations to recruit and randomise, and deliver the intervention to, given the financial and time restraints imposed, but we acknowledge that we are underpowered for station-level outcomes. The number of incidents analysed was constrained by the number of incidents reported to NYP and attended by at least one officer from a participating trial station in the month from mid-November to mid-December 2016. To overcome challenges associated with contamination and sample size, future trials may wish to considerr using a stepped wedge design [20]. In stepped wedged cluster RCTs, clusters are randomly allocated to crossover to the intervention at different time points, with all clusters receiving the intervention eventually [20]. Some of the contamination in our trial was due to movement of police officers between stations. Therefore if future studies were to adopt a stepped wedge design with the police force as the unit of allocation and analysis, some contamination could be avoided. This would be particularly beneficial, for policy-driven interventions which are to be implemented throughout the police force (e.g. street triage) as the design enables a phased roll-out and robust evaluation.

## Conclusion

We found no evidence that a one-day bespoke mental health training package delivered by mental health professionals to frontline officers affected the number of incidents reported to the police control room up to six months after the intervention delivery, but it may have a positive effect on how officers are recording incidents involving individuals with mental health problems. Our trial has demonstrated that trials within the police setting are possible and has highlighted that given the amount of routinely collected police data further research should be conducted in this setting. Researchers should consider issues of contamination and the difficulties of obtaining reliable and meaningful outcome measures. A follow-up of at least one year is also recommended to allow any changes to be detected when undertaking further research in the police setting.

## Supporting information

S1 File Key terms. (DOCX)

S2 File Protocol. (DOCX)

S1 Table Summary of intervention details using the items on the TIDIER checklist. (DOCX)

S2 Table CONSORT checklist. (DOCX)

## Abbreviations

ASB: Anti-social behaviour
CI: Confidence Interval
CIT: Crisis Intervention Team
CoP: College of Policing
HBPoS: Health Based Place of Safety
IRR: Incidence Rate Ratio
IT: Information Technology
MH: Mental Health
MHA: Mental Health Act
NYP: North Yorkshire Police
OR: Odds Ratio
OIC: Officer in Case
PC: Police Constable
PCSO: Police Community Support Officer
PoS: Place of Safety
PSW: Public Safety and Welfare
RCT: Randomised Controlled Trial
RMU: Resource Management Unit
SNC: Safer Neighbourhood Command area
TEMPO: Training and Education about Mental Illness for Police Organisations
USA: United States of America
UK: United Kingdom
YTU: York Trials Unit
