# Supplementary material for: Effectiveness of a training program for police officers who come into contact with people with mental health problems: A pragmatic randomised controlled trial
Source: PLoS One. 2017 Sep 8;12(9):e0184377. doi: 10.1371/journal.pone.0184377 (PMC5590916; doi:10.1371/journal.pone.0184377)
Supplement: S1 File — (DOCX) [file pone.0184377.s001.docx]

**Niche:** Local (NYP) police records management system, used to manage the post-incident investigation of cases. Links details of people, addresses, vehicles, intelligence etc.

**Police constable (PCs):** Officers who respond to 999 calls, investigate volume crime or take initial action at critical incidents. Constables also work within neighbourhood teams to target long term problems [12].

**Police Community Support Officer (PCSOs):** Members of the Safer Neighbourhood Policing Team, working within communities [NYP personal communication 2016]. PCSOs have different roles in different forces including: dealing with minor offences, early intervention to deter people from committing offences, support for front-line officers, house-to-house enquiries, guarding crime scenes, crime prevention advice [13].

**Police Inspector:** Senior operational officers who oversee responses to crucial incidents and all officers on duty [12].

**Police Sergeant:** Supervise teams of officers, oversee police operations, volume crime investigations, demand management issues and take initial control of critical incidents. Typically at least 2-3 sergeants in each borough are on duty at any time [12].

**STORM**: Police (NYP) command and control system, used to record the initial details of an incident and the policing response.
