# Supplementary material for: Effectiveness of a training program for police officers who come into contact with people with mental health problems: A pragmatic randomised controlled trial
Source: PLoS One. 2017 Sep 8;12(9):e0184377. doi: 10.1371/journal.pone.0184377 (PMC5590916; doi:10.1371/journal.pone.0184377)
Supplement: S2 File — (DOCX) [file pone.0184377.s002.docx]

# STUDY PROTOCOL

## PROTOCOL TITLE:

**Effectiveness of a training programme for Police Officers who come into contact with people with mental health problems: a pragmatic randomised controlled trial.**

VERSION: 6.2

DATE: 26^th^ May 2016

## FUNDER

The Police Knowledge Fund, funded by: The Home Office, The College of Policing and the Higher Education Funding Council for England.

## ETHICS COMMITTEE

University of York, Health Sciences Research Governance Committee.

**TRIAL MANAGEMENT**

**The trial team**

The Chief Investigator for the trial is Professor David Torgerson, Director, York Trials Unit, Department of Health Sciences, University of York.

Professor Catherine Hewitt, Deputy Director and Senior Statistician, York Trials Unit, Department of Health Sciences, University of York is the custodian of the quantitative data generated by the study.

Professor Martin Webber, Professor of Social Work, Department of Social Policy and Social Work, University of York is responsible for the development and implementation of the training intervention. He is also custodian of the qualitative elements of the data collected.

Other trial team members (in alphabetical order) are:

Alison Booth, Research Fellow, York Trials Unit (Programme Manager)

Arabella Clarke, Research Fellow, York Trials Unit (Trial Coordinator)

Adwoa Hughes-Morley, Research Fellow, York Trials Unit

Caroline Fairhurst, Statistician, York Trials Unit

Dr Catriona McDaid, Senior Research Fellow, York Trials Unit

Dr Nicola Moran, Research Fellow, Department of Social Policy and Social Work (Training Coordinator)

Inspector Bill Scott, Mental Health Partnership Development Inspector, Partnership Hub, North Yorkshire Police

Mark Richardson, Training Manager, Training Services, North Yorkshire Police.

Kat Chatterton, York Trials Unit, provides secretarial support for the trial.

## BACKGROUND AND RATIONALE

Police Officers fulfil many different roles: maintaining law and order, preventing offences, bringing offenders to justice, and protecting life and property. This means close contact with the general public including people with mental health problems [Her Majesty’s Inspectorate of Constabulary, 2013]. The joint Association of Chief Police Officers/National Policing Improvement Agency/Department of Health guidance for responding to people with mental ill health or learning disabilities recognises that Police Officers and staff are often the first contact for deciding on the nature of appropriate care (e.g. criminal justice or healthcare) [National Policing Improvement Agency, 2010]. The report states that the Police “cannot and indeed are not expected to deal with vulnerable groups on their own”. The Police are signatories of the Mental Health Crisis Care Concordat, a multi-agency initiative actively working on improving outcomes for people experiencing mental health crisis [Department of Health and Concordat Signatories, 2014]. The concordat also recognises the pivotal role played by the Police in identifying and deciding on the most appropriate course of action or referral in individual situations. It is therefore essential that people with mental ill health or learning disabilities are recognised and Police Officers are equipped to assist from the very first point of contact.

Section 136 of the Mental Health Act 1983 gave constables the power to remove from any public place a person who “appears to him to be suffering from mental disorder and to be in immediate need of care or control” and take them to a place of safety [Mental Health Act 1983]. Police forces and individual officers are also required to record information and collect data on incidents involving people with mental ill health or learning disabilities [National Policing Improvement Agency, 2010].

However, Police Officers are not, nor should they be, experts in mental ill health, but they often have to manage difficult and complex problems with insufficient training [Mental Health Cop, 2016]. A joint review by Her Majesty’s Inspectorate of Constabulary, Her Majesty’s Inspectorate of Prisons, the Care Quality Commission and Healthcare Inspectorate Wales found gaps in knowledge around Section 136 [Her Majesty’s Inspectorate of Constabulary, 2013]. Staff were not always aware of responsibilities, even when attempts had been made to provide guidance for officers. The review identified online learning as the main form of training tool for Police Officers; but there were variations in who had completed the training. In field work, the Inspectorate review team said staff they spoke to would value multi-agency, face-to-face training [Her Majesty’s Inspectorate of Constabulary, 2013].

The Independent Commission on Mental Health and Policing reported there is often poor performance in dealing with mental health issues because of the inadequacy of systems and procedures [Independent Commission on Mental Health and Policing, 2013]. Even when a person is identified as having a mental health vulnerability, the information may not be recorded in a coherent manner and there are failures in making use of available resources (e.g. mental health street triage; mental health crisis teams) [Her Majesty’s Inspectorate of Constabulary, 2013; Independent Commission on Mental Health and Policing, 2013].

## AIMS AND RESEARCH QUESTION

The research question being addressed is: What is the effectiveness of a face-to-face mental health training intervention delivered by mental health practitioners to Police Community Support Officers (PCSOs), Police Constables (PCs), Sergeants and Inspectors compared with ‘business as usual’ routine training? (see Appendix 1).

The trial aims to investigate whether the specialised training intervention better equips front line officers to deal with individuals with mental ill-health and reduces demand on police resources. A range of metrics will be used to assess the effects of the training, but the primary measure will be the incidence of calls identified as having a mental health related element received by the North Yorkshire Police (NYP) control room.

The trial also aims to assess the impact of the training intervention on officers’ knowledge of, and attitudes toward, mental ill health and vulnerability; and their confidence in identifying mental distress, responding to people experiencing mental distress, recording incidents as appearing to involve a person experiencing mental distress, referring individuals to local mental health services and any other relevant agency, and working with partner agencies.

Amongst those officers who receive the mental health training, we would expect: knowledge of mental ill health to improve; attitudes toward people experiencing mental ill health to improve; confidence in working with people with mental ill health, referral routes and multi-agency working to increase; and perceived gaps in knowledge and training around mental health to decrease.

## STUDY DESIGN

The study will be a pragmatic cluster randomised controlled trial (RCT) with equal randomisation. Clusters will be police stations allocated to one of two arms (Figure 1):

- Specialised Mental Health Training (intervention group)
- Routine training (control group)

The police stations will be randomised for their officers to receive either the mental health training package or routine training. This design will enable us to examine the effectiveness of the specialised mental health training package. Appendix 2 outlines the trial timelines.

A cluster randomised trial is the best design for minimising contamination between police officers and police stations. Individually randomising police officers is not appropriate as those in the control arm may be partially exposed to the intervention, through interaction with officers receiving the intervention. This was considered a particular risk as police officers often work in pairs and so may have interacted and discussed the intervention or training with one another. Randomising police stations at the cluster level, may also reduce the effects of contamination, as there is much less interaction between officers at different stations.

The trial has been designed to minimise all potential threats to internal validity, such as selection bias and a range of post randomisation bias [Cook and Campbell, 1969; Shadish, Cook and Campbell, 2002; Torgerson and Torgerson, 2008]. In this way, unbiased estimates of the impact of the intervention will be provided. The trial will be reported in line with CONSORT standards [Schulz, 2010].

### PARTICIPANTS AND SETTING

The mental health training intervention will be delivered to front line officers allocated to the intervention group. The Mental Health Partnership Development Inspector for NYP, in conjunction with the NYP Human Resources Department identified that response and safer neighbourhood team officers within the ranks PCSO, PC, Inspector and Sergeant should be included in the trial, as these officers have regular contact with individuals with mental ill health. Definitions of the roles and responsibilities of included officers are provided in Appendix 1.

NYP consists of 39 police stations, which operate within six ‘Safer Command Areas’. Within each of the six Safer Command Areas the two stations with the highest numbers of frontline police officers will be randomised to receive either the specialised mental health training package or usual training (Figure 1). The two largest stations within each of the six Safer Command Areas were selected for the following reasons:

- It was not considered feasible for 50% of NYP stations to receive the intervention within the project time frame (see Appendix 2).
- Smaller/lower tier stations have fewer staff and irregular opening hours. Given that individuals with mental ill health often call the police outside office hours (9am-5pm), there were concerns that any effect of the intervention could be missed within these areas.
- There is greater movement of officers within smaller/lower tier stations. The small numbers of staff operating within smaller/lower tier stations means that if those two officers were not available (e.g. through sickness) officers from larger stations (i.e. York) would be sent to cover this deficit. It was therefore felt that including smaller stations would bring significant contamination risks, due to the more frequent movement of officers to cover smaller stations.

Research team members from the department of Social Policy and Social Work (SPSW) at the University of York will liaise with the NYP training department to recruit the 12 identified police stations to the trial in February 2016. SPSW have prepared trial documentation which NYP will forward to the relevant stations and officers: an information sheet for officers and stations recruited to the trial; information introducing the pre-post survey; and an information sheet and consent form for officers who are approached to take part in an in-depth interview following the second round of data collection (see Appendices 4-9). The training department at NYP will inform relevant officers (PCSOs, PCs, Sergeants and Inspectors) at the stations randomised to the intervention group about the study and arrange release of the relevant officers to attend the training (see Appendix 3).

All officers across the 12 randomised stations will be asked to complete an online survey distributed via the NYP training department before and approximately six months after the delivery of the training (see Appendices 4-9). Qualitative interviews will also be conducted with a sample of 20 frontline officers who received the Specialised Mental Health Training intervention approximately six months after the training (see Appendices 10 and 11).

The NYP training department may choose to withdraw police stations from receiving the specialised training package in favour of the routine training. Such decisions will be communicated to, and recorded by, the Trial Coordinator at the York Trials Unit (YTU). In the scenario where a police station withdraws from providing survey data, data will be retained for all police stations up to the date of their withdrawal and will be used in the analysis, unless specified otherwise by NYP, in which case their details will be removed.

All aspects of the project (RCT and evaluation elements) have been approved by the NYP Training Commissioning Group and so participation in the trial for PCSOs, PCs, Sergeants and Inspectors has been deemed mandatory by the NYP Training Commissioning Group for officers randomised to the intervention group. The intervention will be delivered between May and July 2016 (see Appendix 2). The key phases of the study are outlined in a CONSORT flow diagram (Figure 2).

Figure 1 Identification of participating police stations

Randomised (n=12)

Intervention (n=6)

Control (n=6)

Largest 2 stations

Largest 2 stations

Largest 2 stations

Largest 2 stations

Largest 2 stations

Largest 2 stations

Safer Command Area 1

Safer Command Area 2

Safer Command Area 4

Safer Command Area 3

Safer Command Area 5

Safer Command Area 6

NYP stations (n=39)

Safer Command Areas (n=6)

Eligible Police Stations = 12

Losses=

♦  Declined to participate (n= )

♦  Other reasons (n= )

Randomised =

6 month analysis - Mental health training group

Police stations analysed =

Police stations not analysed (give reasons) =

6 month analysis - Business as usual control group

Police stations analysed =

Police stations not analysed (give reasons) =

Follow-up survey

Lost to follow-up (give reasons) =

Discontinued intervention (give reasons) =

Follow-up survey

Lost to follow-up (give reasons) =

Discontinued involvement (give reasons) =

Baseline survey

Mental health training group =

Business as usual control group=

Figure 2 Consort diagram detailing progression through the trial

### RECRUITMENT

### INCLUSION CRITERIA

Response and safer neighbourhood team officers within the ranks PCSOs, PCs, Inspectors and Sergeants will be included in the trial, as these officers have regular contact with individuals with mental ill health. The two police stations with the highest number of eligible frontline police officers within each of the six Safer Command Areas will be recruited into the trial. The figures for each of these stations were manually extracted from NYP’s in-house ‘duties’ system by the Mental Health Partnership Development Inspector for NYP, who also provided details of the rank and numbers of frontline officers, within each NYP station. The same Inspector in conjunction with the Police Superintendent, Head of Partnership Hub, NYP approved the final numbers and stations to be included in the trial.

### EXCLUSION CRITERIA

To reduce the possibility of contamination, frontline officers with specialist roles (Fire-arms, Major Crimes and Dog units) will be excluded from the trial as these individuals often move between police stations. Control room and custody staff will also be excluded, as the study is aimed at frontline staff.

### RANDOMISATION

Randomisation will occur at the cluster level. An independent statistician at the York Trials Unit will randomise police stations to receive either the intervention or control condition on a 1:1 basis. Minimisation will be used to ensure that the groups are balanced overall in terms of specified covariates using a dedicated computer program, MinimPy [Torgerson & Torgerson, 2007; Saghaei, 2011]. In this instance the groups will be balanced in terms of: cluster size, geographical area and Street Triage (see Appendix 1). Street Triage is in operation within three of the six safer command areas. As this involves close collaboration between police and mental health services, how frontline staff respond to incidents involving mental health may differ in these areas.

## BLINDING

It is not feasible to blind police stations and individual participants to which group they have been allocated. Members of the study team responsible for statistical analysis of the study will be kept blind to each station’s group allocation.

## INTERVENTION

The content and delivery of the training intervention will be developed in close consultation with mental health practitioners from Tees, Esk and Wear Valleys NHS Trust (TEWV) and North Yorkshire Police. The content of the training will be informed by the College of Policing (CoP) Learning Standards, which provide a framework for mental health training for police officers, as well as by findings of the systematic review of evaluations of mental health training for non-mental health professionals being undertaken by the York Trials Unit. The CoP Learning Standards modules include one for all police officers and another targeted at first responders. They are designed for training to be adapted to local police force needs; taking into account existing training on mental health and any other specific local considerations. SPSW in developing the training intervention are therefore taking account of the CoP Learning Standards, existing training, and also ensuring the package meets the requirements of NYP, as outlined in the initial project bid. Thus the training to be delivered to front-line officers in NYP must enhance officers’ understanding of, and ability to:

- ***Identify*** mental vulnerability;
- ***Record*** relevant information using available systems;
- ***Respond*** using appropriate internal and external resources;
- ***Refer*** vulnerable people into services to provide longer-term assistance;
- ***Review*** incidents to make sure that risks have been effectively managed.

The training aims to reduce demand on police resources through improving officers’ knowledge, awareness and understanding of mental ill health and vulnerability, referral pathways (including knowing who to call and when), and the skills necessary to work both with people in mental health distress and with colleagues in partner agencies.

The learning objectives are that by the end of the training officers will be able to:

- Identify if somebody appears to be in mental distress;
- Have the confidence to request that STORM MENTAL HEALTH Tag and Warning Markers be applied in the police records management system Niche and to the Police National Computer (PNC) when believed to be appropriate at the time;
- Recognise / understand different ways of communicating with people experiencing mental distress;
- Evaluate different courses of action to determine which might be the most appropriate, considering the best interests and associated risks for the person experiencing mental distress;
- Have increased understanding of, and confidence in making, referrals (e.g. when, where, who, how);
- Better understand the role/remit/work of other relevant agencies, and have the confidence to work effectively with them;
- Understand the importance of reviewing actions taken, and managing the residual risks, in response to people experiencing mental vulnerability.

The training will be a one-day event, repeated on approximately 25 days to ensure all officers within the ranks of PCSO, PC, Sergeant and Inspector from Response and Safer Neighbourhood Teams from the police stations randomised to the intervention group are able to attend. The individuals delivering the face-to-face training in classroom settings will all be qualified and experienced mental health professionals. It will include:

(a) The opportunity to gain some insight into the real-world experiences of people with different mental health conditions through short video clips of individuals talking about their experiences;

(b) Some traditional classroom style delivery of information, for example around the need to record incidents involving people with mental ill health (knowing who to contact to request that warning markers or tags be placed against individuals or incidents), and around the possible implications both of recording and of failing to record incidents involving people with mental health problems;

(c) Skills based learning around how to communicate with people in mental distress;

(d) working through scenarios in small groups to look at different approaches to engaging and communicating with people in mental distress, and providing opportunities for officers to discuss real-world incidents (anonymized) and scenarios, and seek advice and information from mental health professionals;

(e) Exploration of the types of mental health support services and organizations that exist locally (tailored to each local area);

(f) ‘Talking heads’ video clips of individuals from a range of partner agencies describing the remit of their service/organization and what they can and cannot do; and

(g) Discussion and skills-practice around the importance of reviewing actions taken to ensure that the best interests of the person experiencing mental distress have been paramount.

## DATA COLLECTION

### ROUTINELY COLLECTED DATA

All calls made to NYP are automatically recorded on the in-house IT system (STORM) and are then transferred to the Niche system (see Appendix 1). Data on all calls made to NYP will be collected from the Niche system before (April 2016) and approximately six months after the delivery of the training (duration of follow-up will be confirmed in the statistical analysis plan, once a more detailed understanding of the nature of the data is obtained). Call data will be pseudonymised by the data analyst for NYP and will be securely transferred to the trial statisticians at YTU.

### PARTICIPANT REPORTED DATA

Immediately following the training course, officers will also be asked to complete a feedback form (see Appendix 12). This paper form will ask officers to rate their satisfaction with the course, and their views on the relevance, clarity, usefulness etc. of the course on a 7-point Likert scale. This form is an adaptation of a form used by the College of Policing to evaluate training in a different study.

All officers across the 12 randomised stations will be asked to complete an online survey distributed via the NYP training department before and approximately six months after the delivery of the training (see Appendices 4-9). The survey will explore officers’ knowledge of, and attitudes toward, mental ill health (using the Mental Health Knowledge Schedule (MAKS) standardised questionnaire [Evans-Lacko et al, 2010]); self-reported levels of confidence in recognising the signs and symptoms of mental health conditions, recording incidents as involving issues around mental health, responding to incidents involving people experiencing mental ill health, referring to local specialist mental health services, working with partner agencies in cases involving people with mental ill health, reviewing actions taken in relation to incidents involving mental ill health, and understanding the jargon associated with mental health issues [adapted from Brooker and Sirdifield, 2009]; responses to scenario-based questions around identification of mental distress, recording incidents as appearing to involve an individual in mental distress, responding to individuals in mental distress, referring to mental health services, partnership working, and reviewing actions taken; working relationships with mental health services; communicating with people experiencing mental distress; and perceived gaps in knowledge and training. The post intervention survey will also ask about the perceived impact of the training on the work of the station, individual officers and people experiencing mental distress (see Appendix 6). An email introducing the survey and encouraging officers to participate will be circulated by the NYP Deputy Chief Constable a day or so in advance of the survey being distributed (see Appendix 7). The research team will then ask NYP to circulate an email to all officers that includes information about the survey (prepared by the research team, see Appendix 5) and the link to the survey (see Appendix 6). One week later the research team will ask NYP to circulate a reminder email, including the link to the survey. A further week later NYP will circulate a reminder email from the Deputy Chief Constable (see Appendix 8). A day or two later the research team will ask NYP to circulate a final reminder email, again containing the link to the survey.

The survey will be piloted prior to distribution to officers involved in the trial. Piloting of the survey will take place with volunteer front-line officers of the ranks PCSO, PC, Sergeant and Inspector from a different police force, with access facilitated by the Mental Health Partnership Development Inspector from NYP. Officers who take part in the piloting of the survey will be asked to comment on both the format and content of the survey, and on the time required to complete the survey. Any issues revealed during the pilot stage will be addressed prior to the distribution of the survey to NYP police officers.

The survey forms part of the evaluation of the RCT, but also will be used to evaluate two of the other work streams: (i) the work stream on research methods training for senior officers with the aim of increasing evidence-based policing; and (ii) the overall evaluation of the impact of involvement with the Connect project on all police officers and police staff within NYP. The online surveys (pre and post) will include five question sets: basic demographic information; knowledge of, and attitudes toward, mental ill health; outcome measures for the RCT; partnership working and referral pathways between the police, mental health services and other statutory and third sector organisations; and the use and understanding of evidence based research. All police officers and police staff will be asked a small number of questions about their demographic data, their awareness of mental ill health, and current practices within NYP around working with people in mental distress and collaborative working with partner agencies (sections A, B and D of the survey). Those who took part in the RCT will be asked an additional set of questions (section C of the survey), while those in ranks targeted for the research methods training will be asked a different set of additional questions (section E of the survey). The survey will be set up using Qualtrics software that will direct officers to different sets of questions depending upon their area(s) of involvement with the project (this will be based upon their rank and, for those involved in the RCT, the station to which they report).

In addition, a sample of 20 front-line officers who received the training intervention will be interviewed approximately six months following the training to explore in greater depth the areas covered by the survey, issues coming out of the survey and feedback from the training sessions. The topic guide for the interviews will be developed over summer 2016 and will be piloted with two officers who received the training, and adapted in light of any issues identified. We will ensure that the post-pilot topic guide is presented to the HSRGC in advance of interviews commencing.

We anticipate that a sample size of 20 officers will be sufficient to reach theoretical saturation. The sample will be drawn from those officers who completed both the pre and post intervention surveys and who indicated in the post intervention survey that they were interested in finding out more about taking part in a follow-up interview. We will recruit officers through other methods, such as an email request, if fewer than 20 eligible officers volunteer. If more than 20 officers meet the criteria, SPSW will develop a sampling frame to include officers:

- from stations of different sizes;
- from stations in different geographical locations (to reflect such differences as the number and reach of local mental health services);
- from areas with and without street triage schemes (i.e. with different working relationships with mental health practitioners);
- with different self-reported levels of understanding and confidence in working with people with mental ill health, mental health services and partnership working; and
- reporting different levels of impact of the training on the work of their station, their own day-to-day work and people with mental ill health.

An information sheet outlining the purpose of the interview and what taking part would entail will be emailed to police officers in the sample via the NYP human resources department (see Appendix 9). Officers interested in taking part in an interview may then telephone or email the researcher directly to arrange a mutually convenient time and date for the interview, which will take place at the officer’s local police station. The interviews are scheduled to take place between December 2016 and January 2017. A consent form will be completed prior to commencement of the interview (see Appendix 10).

## OUTCOME MEASURES

The primary outcome measure will be the number of telephone calls which result in a police response received by the North Yorkshire Police (NYP) control room. This outcome measure will be analysed at the person-level, where each person is a member of the public who makes at least one call to the control room which results in a police response (known as a caller). For analysis, each caller will be associated with the police station in which the attending/responding police officer(s) works. If an individual has responses from both intervention and control officers within the data extraction window the chronologically first incident will be used to assign the individual to the intervention or control group. Secondary outcomes also routinely collected via the Niche system include: number of incidents with section 136 applied; number of incidents with mental health tags applied; number of individuals with a mental health flag applied resulting from each call; and number of frequent callers. The appropriateness of mental health tags applied to a random sample of incidents will be checked by an independent mental health professional. Other secondary outcomes will be measured at the level of the police officers from participating police stations and include: MAKS score; police officers’ satisfaction with the course, and their views on the relevance, clarity, usefulness etc. of the training; and self-reported levels of confidence in recognising and managing mental health conditions.

## SAMPLE SIZE CALCULATION

Our pragmatic cluster trial will involve approximately 1160 frontline officers from 12 clusters. At least 4 clusters per arm are recommended for a cluster RCT [Hayes 2009].  Our sample size exceeds this minimum recommendation whilst allowing for resource constraints and remaining sufficiently large to provide meaningful information.

## ANALYSIS PLAN

***Analysis of quantitative data***

A detailed statistical analysis plan for the analysis of quantitative data will be written and signed off by the Trial Management Team prior to data analysis, but analyses are described in brief below. Analysis will be conducted using the principles of intention to treat (ITT). All NYP stations and officers will be analysed in the group to which they were randomised, irrespective of whether or not they actually received, or adhered to, the intervention. Statistical significance will be assessed using two-sided tests at the 5% level unless otherwise stated. Analyses and results will be reported in accordance with the CONSORT extension for Cluster Trials (Campbell et al, 2012).

Summaries of the characteristics of the police stations and officers (where available) will be presented by trial arm. Continuous measures will be summarized using descriptive statistics (mean, standard deviation, median, minimum and maximum) and categorical data will be reported as counts and percentages. No formal statistical comparisons by trial arm will be undertaken.

The primary analysis will use a mixed effects Poisson (or negative binomial) regression model as appropriate to investigate the number of calls with a response per caller. The model will be adjusted for the police station level covariates of group allocation, average number of calls per person with a response per station at baseline, and the minimization factors as fixed effects, and for police station as a random effect. The incidence rate ratio for the intervention versus control group will be presented with a 95% confidence interval (CI) and p-value.

The likelihood of an incident having section 136 applied will be analysed at the incident level using a mixed logistic regression model adjusted for the police station level covariates of group allocation, proportion of incidents with section 136 applied at baseline, and the minimization factors as fixed effects, and for police station as a random effect. The odds ratio for the intervention versus control group will be presented with a 95% confidence interval (CI) and p-value. A similar approach will be taken for the outcome of number of incidents with mental health tags applied; however, data permitting, the model will be adjusted additionally for attending officer characteristics (such as average years in service, male/female/mixed, average age).

The number of individuals with a mental health flag applied for each call with a response will be analysed in a similar way to the primary outcome, with an additional covariate for the number of officers in attendance at the incident.

Frequent callers will be identified and linked to a station either by having at least one response by a participating officer in April 2016 from that station or by the location of the call. The number of calls for each frequent caller per month will be collected for the time period between between April 2015 and December 2016/January 2017 dependent on when the outcome is collected. Summary statistics will be produced and an interrupted time series analysis will be conducted (if possible) potentially accounting for multiple interventions, to investigate whether there is evidence of a positive effect of the current investigation.

A random sample of all incidents (approx. 100) will be independently reviewed by a Mental Health professional to assess whether a tag should have been applied to the incident (Yes/No). We will assess the agreement of this assessment with the actual application, or not, of a tag, and descriptive statistics will be presented by arm at the level of the police station.

Analysis of MAKS and other quantitative scores from the online survey, and of officer satisfaction with the training will be via appropriate regression techniques for continuous or categorical data adjusting for group allocation, minimisation factors, pertinent baseline characteristics of the officers (data permitting), baseline value of the outcome (for pre-post survey outcomes), and police station as a random effect.

***Analysis of open-ended survey responses***

Open-ended survey questions will be coded and analysed thematically using content analysis. The coding frame will be developed based on identified themes, informed by the research questions, but with the flexibility to accommodate additional emerging themes and issues. Analysis of this data will be reported alongside the analysis of the quantitative survey data, and will help to explain or critically interrogate the quantitative findings.

***Analysis of qualitative interviews:***

Semi-structured interviews with a sample of 20 front-line police officers who attended the mental health training will be recorded, with consent, and transcribed verbatim. If consent is not given to record interviews, the researcher will take detailed written-notes. The Framework method [Ritchie and Lewis, 2003] will be used to analyse the interview data. Framework is a matrix-based method for ordering and synthesising data into an analytical hierarchy of key themes, concepts and categories. The analysis will follow the five stages of framework analysis: familiarisation, identifying conceptual themes, indexing, charting and mapping. A thematic framework will be developed through a process of reading transcripts and detailed interview summaries and discussions within the team. The framework will include a priori themes (reflected in the topic guide) and emergent themes. The data will be summarised under these themes on a series of charts (one per theme) and indexed back to the location in the raw data. The framework has transparency, it allows the researcher to move between levels of abstraction without losing track of the raw data, and to test emerging findings within the wider context of the interviews. Charts will be created using Excel. Each row on a chart will be used exclusively for one interviewee. The far left hand column on each chart will be used to record background information about participants (ID, rank, role). The identification of themes and analysis of the charted data will be an iterative process with ongoing discussion within the research team. Quotations from interviewees will be used to provide evidence for the conclusions drawn.

## TRIAL SCHEDULE

The trial schedule can be found in Appendix 2.

**CONSENT**

The delivery of the mental health training intervention has been approved by the Training Commissioning Group within NYP. As per usual practice, for training approved through these channels, undertaking the specialised mental health training will be mandatory for officers (PCSOs, PC, Inspectors and Sergeants) in stations randomised to the intervention group. The NYP training department will abstract all relevant officers from their duties to enable them to undertake the training. To facilitate the provision of cover for their shifts, the training will be delivered over a period of three months (May to July 2016). The training manager for NYP will provide police stations and officers taking part in the trial with information sheets (see Appendix 3-4) about the trial and about the survey. The information sheet will also be used as the email to officers introducing the survey and provide the link to the survey. Participation in the survey will be voluntary. The front page of the survey includes a statement of consent and a box for participants to select if they wish to take part. The survey will only begin if the consent box is selected.

Participation in completion of the follow-up questionnaires will also be voluntary (see Appendix 5). The information sheet for the survey will also serve as the front page of the online survey.

Following the second round of data collection (the post-intervention survey that will be circulated approximately six months following delivery of the training), the research team will identify a sample of officers from the intervention group who received the training, completed both pre and post surveys, and indicated in the post survey that they were willing to be contacted to find out more about taking part in a follow-up interview. This sample of officers will be sent an additional information sheet (see Appendix 9) outlining what the interview would entail. Those who agree to take part would be asked to complete a consent form prior to interview (see Appendix 10). All trial documentation (i.e. information sheets, consent form, survey) will also be provided to a senior officer at each of the randomised stations.

## ETHICS REVIEW

This protocol and all associated trial documents (i.e. information sheets, consent form, survey etc.) will be submitted to the University of York, Health Sciences Research Governance Committee (HSRGC) for ethics and governance approvals.

## UNIVERSITY INDEMNITY

The University of York Insurance office has been informed of this trial and a copy of the ‘automatic’ public liability insurance cover certificate requested. Cover is automatic if the research is within the UK and limited to questionnaires and interviews with police officers (YTU SOP T05 University of York Public Liability Insurance for Research V1.0, 30 April 2014). Responsibility for obtaining University indemnity has been delegated to the Trial Coordinator.

## ANTICIPATED RISKS

The study does not involve any invasive or potentially harmful procedures and is therefore considered low risk. However, the training package focuses on how officers respond to incidents involving individuals in mental health crisis or distress. These are sensitive issues. With an estimated one in four of the population experiencing mental ill health at some point in their lives, we would expect a large number of officers to have experienced mental ill health personally or amongst their family, friends or colleagues. The training will be delivered by mental health professionals who will not only be experts in discussing the issues sensitively but will also encourage and foster a supportive environment in the training sessions. In addition, a support information sheet (see Appendix 11) will be included in the resource packs provided to officers at the end of the training sessions and will also be provided to officers following the qualitative interviews. The support information sheet contains details and contact details of organisations that officers could contact for information, advice or emotional support. Should any of the trainers have concerns about individuals during the training, they will speak to the officer confidentially at the first available opportunity (e.g. break) to assess the situation, arrange a quiet space for the officer to sit out some of the training until they feel able to continue or to leave the training altogether if appropriate. The trainer will spend as much time as possible with the officer but may need to call for support from a colleague, senior officer, family member or friend of the officer.

If an officer appears distressed during an interview, the interview will be paused, the situation assessed, and, if the officer feels it necessary, the interview will be terminated. The researcher will stay with the officer for a length of time to assist, and may call for support from a person of the officer’s choosing (colleague, senior officer, family member, friend). The officer will have final say over whether the interview data is included in the project.

Our procedure for dealing with a disclosure of harm is in the process of being set up with NYP. However, our proposed course of action is:

- If a disclosure of harm is made during the interview, the interviewer must explain that someone else will need to be told. This will be the Police Officer’s superior officer who will then take responsibility for any further action.
- Participants will be reminded of the disclosure policy at the start of the interview.
- The participant will be kept informed at all times as to who has been told.

Additional low risks associated with the project are outlined below:

*Recruitment of police stations:* whilst undertaking the mental health training will be mandatory for those officers in stations randomised to the intervention group, the NYP training department may withdraw stations from the trial. YTU and SPSW will work with NYP to minimise this risk.

*Attrition and loss to follow up:* it is essential that this is kept to a minimum. YTU and SPSW will work jointly with NYP to help minimise attrition.

*Contamination:* Whilst movement of frontline officers among stations within a Safer Command Area is minimal, there are instances where this is necessary. For instance, should one of the stations within each Safer Command Area not have the required minimum number of staff on duty, officers from other stations within that Safer Command Area are sent to cover this shortage. By including the two largest stations within each of the Safer Command Areas, the effect of this movement and potential for contamination is reduced as it is unlikely that any of the larger stations would not have the required number of staff on duty at any one time.

## PUBLICATIONS AND DISSEMINATION

A project specific public website is being built and will be used as a vehicle for raising awareness of the Connect project aims, methods, progress and results, including this trial. When the trial results are available we will tailor dissemination of the findings to the relevant audiences (e.g. the police, policymakers and academics) and produce appropriate outputs. These may include: a formal report (project book chapter), a summary report in lay language, academic publications, conference presentations, workshops, news releases and short articles/blogs for the websites of interested organisations and other relevant lay media. Social media will also be used to raise awareness of the trial. The trial findings will be presented at a Connect project Partners meeting and shared with the national CoP. Details of the RCT are already available on the CoP Policing and Crime Reduction Research Map <http://whatworks.college.police.uk/Research/Research-Map/Pages/Research-Map.aspx>

The findings will be fed back to NYP to inform the roll out of the intervention training to other officers in NYP and the content and format of future training programmes. In collaborating with the CoP training unit, we anticipate the findings of the RCT will impact on the next review of the National Policing Curriculum for Mental Health and Learning Disabilities, via the CoP’s training maintenance cycle.

## CONFIDENTIALITY

Information provided for the purposes of the study will be kept strictly confidential.

All routinely collected Police trial data will be anonymised by the data analyst at NYP before being transferred to the University of York. Any data shared between researchers will be done following the data protection policy at the University of York. Any electronic data will be stored on a password protected server within the Department of Health Sciences at the University of York.

Participants will be asked for their collar number so as to match up data from the police databases to pre and post survey responses. Nobody outside of the research team will have access to this data. Collar numbers will not be used to identify or target non-responders to the surveys. Researchers will replace collar numbers with ID numbers once surveys have been completed. The data key will be stored separately on a password protected file on a password protected server at the University of York.

All interviewees will be provided with ID codes which will be used during the interviews and write up of the study. No identifiable information, which may cause participants to be identified will be published. All interview data will be recorded on encrypted digital recorders. Interview data will be stored on password protected computers at the University of York. Paper consent forms will be stored separately to interview data in a locked filing cabinet in a locked office in SPSW.

The research team at YTU and SPSW will be the only individuals with access to trial and interview data during the study.

## SECURITY OF TRIAL DOCUMENTS

All study data will be stored and transferred in accordance with the Department of Health Sciences Data Security Policy at the University of York (<https://hswebstaff.york.ac.uk/docs/it/it-info-gov/>). All study data will be stored on a secure server accessed via a password protected computer at the University of York. The secure University Drop Off system or encrypted memory sticks will be used should information require transfer.

All data will be stored for a minimum of five years after the end of the main analysis of the trial. Following trial completion, essential trial documents will be transferred to archive. All storage and archiving will be conducted in line with the YTU Standard Operating Procedure.

Interview data will be psudonymised and stored on a secure password-protected server at the University of York. Consent forms and a key linking IDs to the participants will be kept separately in locked filing cabinets in a locked office in SPSW.

Only members of the trial team will have access to the trial documents and data.

### DATA MANAGEMENT

Trial data will be handled in accordance with NYP policies and the appropriate YTU data management procedures: e.g. DM01 CRF Design; DM03 Manual Entry of Data; DM05 Second Checking of Data.

A data monitoring committee will not be constituted. This is a low risk study involving a one off training intervention involving the use of routinely collected data, and evaluation data collected at baseline and six months post intervention. This makes data monitoring neither feasible nor appropriate. (YTU SOP T04 Data Monitoring Committees V1.0, 25 Feb 2015)

**TRIAL MANAGEMENT GROUP**

The Trial Management Group (TMG) has been constituted in accordance with YTU SOP T02 Trial Management Groups V1.0 15 Jan 2016. Membership consists of the trial team members, with additional people invited to join meetings when relevant or necessary.

Meetings will be held on a two monthly basis as a minimum, but two weekly during the development phase of the trial. Responsibility for calling and organising TMG meetings is delegated to the Programme Manager. Action points from meetings will be recorded and circulated to all Group members and a copy held in the Trial Master File.

The role of the TMG is to:

- Input into and comment on the protocol and other trial documentation at the start of the trial and throughout the duration of the trial should amendments be required.
- Input into the development of the statistical analysis plan.
- Be involved in the day to day running of the trial by supporting the CI and Trial Co-ordinator.
- Input into monitoring the progress of the trial, adherence to the protocol, and the accumulation of new information/ evidence of relevance to the trial.
- Input into funders meetings where required and assist with responses to any issues or concerns these groups may raise.
- Promote the trial.
- Encourage recruitment and provide advice on alternative recruitment strategies.
- Provide advice relating to the trial design and /or conduct where relevant.
- Provide advice on any substantial changes to trial
- Provide advice on publication strategy.
- Input into the interpretation and writing up of the trial results

**TRIAL STEERING GROUP**

As this trial constitutes a research stream within the Connect: evidence based policing project, the Connect Management Group will act as the Steering Group for the trial. The chairman of the Connect Management Group is Professor Martin Smith, Head of the Department of Politics, University of York and Principle Investigator for the Connect project as a whole.

The Chairman and following members of the group are independent of the trial: Superintendent Richard Anderson, Sergeant Denise Wond, and Samantha Pariser, of North Yorkshire Police; Paul Quinton, and Daniel Packham of the College of Policing. Professor Tracey Bywater, Health Sciences, University of York; Jonathan Haslam, Institute for Effective Education, University of York; Professor Jillian Macbryde, Management School, University of York; Josine Opmeer, Research Development Manager, Faculty of Social Sciences, University of York; Nihan Toprakkiran, Carlos Solar and Ed Kirby, Department of Politics, University of York.

The following members of the Trial Management Group also sit on the Connect Management Group: Bill Scott, Martin Webber, Nicola Moran, David Torgerson, Catherine Hewitt, Catriona McDaid, and Alison Booth.

The role of the Connect Management Group as the Trial Steering Group is to:

- Provide overall supervision on behalf of and reporting to the Funders.
- Ensure appropriate ethical and other approvals are obtained in line with the project plan.
- Monitor progress of the trial

The organisation of bi-monthly meetings has been delegated to Rosie Abbotts Connect Project Coordinator, Department of Politics.

**TRIAL MASTER FILE**

The Trial Master File containing all the essential documents relating to the trial will be created and maintained by the Trial Coordinator and securely stored at YTU. The Training Master File containing all the essential documents related to delivery of the training intervention will be created and maintained by the Training Coordinator and securely stored at SPSW. (YTU TO1 Trial Master File SOP (V5.0, 5 Feb 2016)

The trial and training documentation will be stored and archived in accordance with YTU SOP YT03 Storage and Archiving (V2.0, 30 Sept 2014)

## REFERENCES

Brooker, C. and Sirdifield, C. (2009) *An Evaluation of Mental Health Awareness Training for Probation Staff*, University of Lincoln. <http://eprints.lincoln.ac.uk/2517/1/MHATFINAL.pdf>

Campbell MK, Piaggio G, Elbourne DR, Altman DG; for the CONSORT Group. Consort 2010 statement: extension to cluster randomised trials. BMJ. 2012 Sep 4;345:e5661. PMID: 22951546

Cook, T. D., & Campbell, D. T. (1979). Quasi-experimentation: Design and analysis issues for field settings. Boston, MA: Houghton Mifflin Company.

Department of Health and Concordat signatories. Mental Health Crisis Care Concordat Improving outcomes for people experiencing mental health crisis. February 2014.

Evans-Lacko, S., Little, K., Meltzer, H., Rose, D., Rhydderch, D., Henderson, C., and Thornicroft, G. (2010) Development and Psychometric Propoerties of the Mental Health Knowledge Schedule, *Canadian Journal of Psychiatry*. 2010;55(7):440–448.

Hayes RJ, Moulton LH., *Cluster Randomised Trials*. 2009

Her Majesty’s Inspectorate of Constabulary CQC, Her Majesty’s Inspectorate of Prisons, and Healthcare Inspectorate Wales. A Criminal Use of Police Cells? The use of police custody as a place of safety for people with mental health needs. June 2013.

Independent Commission on Mental Health and Policing. Independent Commission on Mental Health and Policing Report. May 2013.

Mental Health Act 1983 Section 136. The Stationary Office. UK: http://www.legislation.gov.uk/ukpga/1983/20/section/136

Mental health cop: a venn diagram of policing, mental health and criminal justice. 2016. Available [online] <https://mentalhealthcop.wordpress.com/2012/03/26/police-rank-and-roles-explained/>

NARPO. National Association of Retired Police Officers. Available at: www.narponorthyorkshire.org

National Policing Improvement Agency on behalf of the Association of Chief Police Officers. Guidance on responding to people with mental ill health or learning disabilities. 2010.

Ritchie, J. and Lewis. J. (eds.) (2003) Qualitative Research Practice: A Guide for Social Science Students and Researchers. Sage Publications, London

Saghaei M. An Overview of Randomization and Minimization Programs for Randomized Clinical Trials. Journal of Medical Signals and Sensors. 2011;1(1):55-61.

Schulz Kenneth F, Altman Douglas G, Moher David.CONSORT 2010 Statement: updated guidelines for reporting parallel group randomised trials BMJ 2010; 340 :c332

Shadish, W. R., Cook, T. D., & Campbell, D. T. (2002). Experimental and quasi-experimental designs for generalized causal inference. Boston, MA: Houghton Mifflin.

Torgerson, C.J. and D.J. Torgerson, The use of minimization to form comparison groups in educational research. Educational Studies, 2007. 33(3): p. 333-337.

Torgerson, D. J., & Torgerson, C. J. (2008). Designing randomised trials in health, education and the social sciences: an introduction. Palgrave Macmillan.

#### Appendix 1

Glossary of terms and list of acronyms

**Niche:** Local (NYP) police records management system, used to manage the post-incident investigation of cases. Links details of people, addresses, vehicles, intelligence etc.

**Police Constables (PCs):** Officers who respond to 999 calls, investigate volume crime or take initial action at critical incidents. Constables also work within neighbourhood teams to target long term problems [Mental Health Cop, 2016].

**Police Community Support Officers (PCSO):** Members of the Safer Neighbourhood Policing Team, working within communities [*NYP personal communication 2016*]. PCSOs have different roles in different forces including: dealing with minor offences, early intervention to deter people from committing offences, support for front-line officers, house-to-house enquiries, guarding crime scenes, crime prevention advice [College of Policing, 2016].

**Police Inspector:** Senior operational officers who oversee responses to crucial incidents and all officers on duty [Mental Health Cop, 2016].

**Police Sergeant:** Supervise teams of officers, oversee police operations, volume crime investigations, demand management issues and take initial control of critical indents. Typically at least 2-3 sergeants in each borough are on duty at any time [Mental Health Cop, 2016].

**STORM:** Police (NYP) command and control system, used to record the initial details of an incident and the policing response.

**Street Triage:** Innovative model of collaboration between police and Mental Health services to provide a rapid response to mentally vulnerable people in contact with police.

**Tag:** Coding used on the initial STORM incident to indicate the type of incident being reported (e.g. Mental Health, Triage, S136, Domestic, Concern for safety)

**Warning Markers:** Risk markers applied to individuals records on Niche (e.g. Mental Disorder, Suicidal, Self-harm, Violent, Ailment, Contagious)

**Acronyms**

COP College of Policing

NYP North Yorkshire Police

PC Police Constable

PCSO Police Community Support Officer

SPSW Social Police and Social Work

TEWV Tees, Esk and Wear Valleys NHS Trust

YTU York Trials Unit

#### Appendix 2

Project Timeline

|  | 2016 | | | | | | | | | | | | 2017 | | |
| --- | --- | --- | --- | --- | --- | --- | --- | --- | --- | --- | --- | --- | --- | --- | --- |
|  | Jan | Feb | March | April | May | June | July | Aug | Sept | Oct | Nov | Dec | Jan | Feb | Mar |
| Submit business case for training intervention |  |  |  |  |  |  |  |  |  |  |  |  |  |  |  |
| Develop intervention |  |  |  |  |  |  |  |  |  |  |  |  |  |  |  |
| Develop evaluation questionnaire |  |  |  |  |  |  |  |  |  |  |  |  |  |  |  |
| Develop information sheet and consent form |  |  |  |  |  |  |  |  |  |  |  |  |  |  |  |
| Apply for University Ethics |  |  |  |  |  |  |  |  |  |  |  |  |  |  |  |
| Recruitment of stations then randomisation |  |  |  |  |  |  |  |  |  |  |  |  |  |  |  |
| Administration and intervention prep (room bookings etc) |  |  |  |  |  |  |  |  |  |  |  |  |  |  |  |
| Pilot Survey |  |  |  |  |  |  |  |  |  |  |  |  |  |  |  |
| Pilot training intervention |  |  |  |  |  |  |  |  |  |  |  |  |  |  |  |
| Obtain anonymised routinely collected data |  |  |  |  |  |  |  |  |  |  |  |  |  |  |  |
| Data collection (survey) |  |  |  |  |  |  |  |  |  |  |  |  |  |  |  |
| Deliver intervention |  |  |  |  |  |  |  |  |  |  |  |  |  |  |  |
| Develop topic guide for qualitative interviews |  |  |  |  |  |  |  |  |  |  |  |  |  |  |  |
| Pilot topic guide |  |  |  |  |  |  |  |  |  |  |  |  |  |  |  |
| Qualitative interviews |  |  |  |  |  |  |  |  |  |  |  |  |  |  |  |
| Follow up-trial |  |  |  |  |  |  |  |  |  |  |  |  |  |  |  |
| Start write up |  |  |  |  |  |  |  |  |  |  |  |  |  |  |  |
| Analysis and write up |  |  |  |  |  |  |  |  |  |  |  |  |  |  |  |

|  | 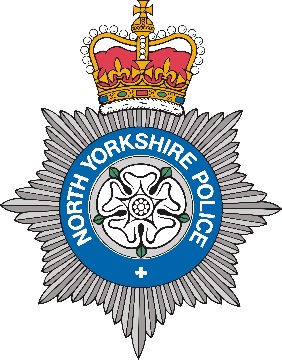 |
| --- | --- |


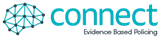

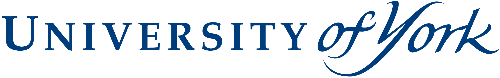
**APPENDIX 3**

**Mental Health Training for Front Line Police Officers: a Randomised Controlled Trial (RCT)**

**Information Sheet**

**Background**

A significant amount of police time is spent dealing with incidents involving people who are mentally vulnerable. Whilst we are acutely aware that police officers are not, and indeed should not be, experts in mental health, they are often the first responders and yet have minimal training in this area. Last year, North Yorkshire Police (NYP) in conjunction with the University of York and the Office of the Police and Crime Commissioner North Yorkshire obtained over £1.1M to work together to develop evidence, research and training with a focus on mental health. The ‘Connect’ project includes a randomised controlled trial of new mental health training for front-line officers; this will build upon the Rethinking Mental Health training being delivered by NYP during early 2016. The training aims to increase awareness and identification of mental health vulnerabilities, improve the recording of incidences involving people with mental ill-health, enhance skills in communicating with people in mental health distress, provide a clearer understanding of referral pathways into mental health services, and aid multiagency working.

**What is a randomised controlled trial?**

In order to measure the effectiveness of the training we are conducting a randomised controlled trial (RCT). Out of 12 identified police stations in North Yorkshire we are selecting six at random to receive the new training. Officers attached to the other six stations will receive any training currently available in NYP. Officers in the ranks of PCSO, Police Constable, Sergeant and Inspector from Response and Safer Neighbourhood Teams in the stations randomised to the intervention group will undertake a one-day training course on working with people with mental health problems in late Spring/ Summer 2016. By analysing outcomes from the two groups before and after the intervention we are able to measure the impact of the training. The trial will explore whether the new training better equips front-line officers to deal with individuals with mental ill-health and reduce demand on police resources.

**Why am I being asked to take part?**

You are attached to one of the police stations taking part in the trial. The training has been approved by NYP and so, as per usual, will be mandatory for identified ranks. If your station has been randomised to the intervention group and you are a Response or SNT PCSO, PC, Sergeant or Inspector, then you will receive the training. The one-day training course will be repeated as many times as is necessary to train all the identified officers. Officers in the control group will receive the training at a later date after the trial has been completed.

**What will the training entail?**

The training will include classroom style delivery of information; skills based learning around communicating with people in mental health crisis; videos of mental health service users talking about their experiences; role play activities; and small group discussions. There will be opportunities throughout the day for officers to discuss real-world incidents (anonymised), and seek advice and information. The content of the training has been developed by Professor Martin Webber and Dr Nicola Moran from the Dept. of Social Policy and Social Work at the University of York, in conjunction with mental health professionals, and in consultation with NYP’s Mental Health Partnership Development Inspector Bill Scott. The training will be a localised NYP version of the mental health training currently being developed by the College of Policing. The findings from the RCT will help to inform the development of College of Policing training on working with people with mental ill health.

**Who will deliver the training?**

Depending upon scheduling, the training will likely be delivered by mental health professionals from across Tees, Esk and Wear Valley NHS Trust, Leeds Community Healthcare NHS Trust, and the Department of Health Sciences at the University of York.

**Who is undertaking the study and how is it funded?**

The project is funded by the College of Policing, the Home Office, and the Higher Education Funding Council for England (HEFCE), through the Police Knowledge Fund. The trial of the mental health training is being undertaken by an interdisciplinary team of researchers from the Department of Social Policy and Social Work and the York Trials Unit at the University of York, in conjunction with NYP.

**Evaluating the training**

In order to identify and measure the impact of the training, it is essential that officers in both the control and intervention groups complete two online surveys – the first will be circulated by NYP in Spring 2016 and aims to collect baseline data; the second will be circulated towards the end of the year and will gather follow-up data. In addition, a number of measures of routinely collected data will be collected at the start and finish of the trial. Comparison of responses at the end of the research period will help us to measure the effectiveness of the training and make recommendations to NYP and the College of Policing to shape future mental health training for front-line officers. The results of the trial (anonymised) will also be presented to the funding body and published in academic and policing journals.

**Confidentiality**

All data will be anonymised, thus nobody taking part will be identifiable in the project report or any other publication. Only members of the research team will have access to the data, which will be kept in locked storage or on password protected computers at the University of York. All information collected during the course of the research will be kept strictly confidential in line with the Data Protection Act. Data will be securely stored for five years from the end of the study and then destroyed.

**Ethical approval**

This randomised controlled trial of mental health training for front-line police officers has been approved by the Health Sciences Research Governance Committee at the University of York.

**Further information**

If you would like any further information on the training please contact Dr Nicola Moran ([nicola.moran@york.ac.uk](mailto:nicola.moran@york.ac.uk)), Dept. of Social Policy and Social Work, University of York, York, YO10 5DD, Tel.: 01904 321246.

|  | 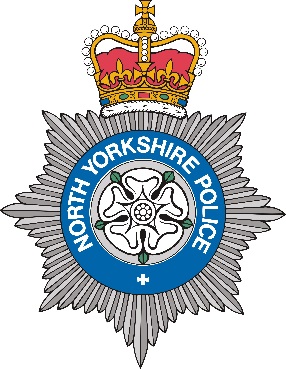 |
| --- | --- |


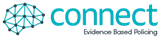

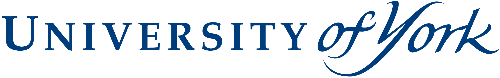


**APPENDIX 4**

**Evaluation of the ‘Connect’ mental health project**

**Information Sheet: Survey 1**

**What is the research about and what does the survey ask?**

In 2015 North Yorkshire Police (NYP) in conjunction with the University of York and the Office of the Police and Crime Commissioner North Yorkshire obtained funding of over £1.1M to work together to co-produce evidence, research and training with a focus on mental ill health and vulnerabilities: the Connect project. The Connect project includes:

- A randomised controlled trial (RCT) of mental health training for front-line officers, developed in conjunction with the College of Policing;
- Training on research methods and using research for middle and senior-ranking officers.

A significant proportion of NYP officers will be involved in one element of the research. It is vitally important that we identify the impact, benefit and outcomes of the training courses for NYP. To help us evaluate the impact of the training – and potentially more subtle impacts of NYP’s involvement in the Connect project – we need officers from all ranks and roles to complete this survey.

The survey has been developed using Qualtrics software that will direct you to different sets of questions depending upon your future area(s) of involvement with the overall project. All police officers and police staff will be asked a small number of questions about their awareness of mental ill health, current practices within NYP around working with people in mental distress and collaborative working with partner agencies. Those who took part in the RCT will be asked an additional set of questions, while those in ranks targeted for the research methods training will be asked a different set of additional questions.

In order to identify and measure the impact of the training we ask that you complete this survey now, so that we can gather baseline data prior to delivery of the training. We will also request that you complete a further survey that will be circulated towards the end of the year to enable us to collect follow-up data. Comparison of the data from both surveys will allow us to analyse the impact of the training and of NYP’s involvement with the Connect project more broadly.

**How long will it take to complete the survey?**

Completing the survey should take no more than 10-15 minutes and will mainly involve ticking boxes. The specific questions you are asked will depend on which element(s) of the research you will be involved with.

**What are the benefits of completing the survey?**

By completing the survey you will help us to measure the effectiveness of the training and this will help us to refine and deliver training that has increased benefit to individual police officers, the force more broadly, and the individuals in mental distress who come to the attention of the police. Understanding if and how the training works will help us to refine it. This is likely to reduce demand on police resources and highlight the best way of allocating resources in the future.

**Who is undertaking the study and how is it funded?**

The Connect project is funded by the College of Policing, the Home Office, and the Higher Education Funding Council for England (HEFCE), through the Police Knowledge Fund. The research is being undertaken by an interdisciplinary team of researchers from the University of York.

**Do I have to take part in the survey?**

No, you do not have to take part in the survey. If you decide not to take part that is OK, you do not have to give a reason. If you want to take part but do not want to answer some of the questions, just leave them blank. Whether or not you decide to take part will not affect your position in NYP; nobody within NYP will be made aware of which officers have chosen not to complete the survey. However, reminders and emails encouraging officers to complete the survey will be circulated to all officers.

**Confidentiality**

All data will be anonymised, thus nobody taking part will be identifiable in the project report or any other publication. Your collar number will be removed from the survey so that all the information is anonymous. Only members of the research team will have access to the data, which will be kept on password protected computers at the University of York. Data will be securely stored for five years from the end of the study and then destroyed. All information collected during the course of the research will be kept strictly confidential in line with the Data Protection Act.

**What will happen to the information?**

Once the fieldwork is complete we will collate and analyse the data to help us determine the effectiveness of the training and of the project as a whole. This will be reported back (in anonymised form) to NYP. The research findings will also be published in report form for the funding body, and will be published in academic and policing journals.

**Ethical approval**

This evaluation of the RCT on mental health training, research methods training, and the Connect project has been approved by the Health Sciences Research Governance Committee and the Social Policy and Social Work departmental ethics committee at the University of York.

**What happens next?**

If you are willing to take the survey please click on this link [link to survey], read the section on consent and check the relevant box. Then click on ‘next’ and the survey will begin.

**Further information**

If you would like any further information on the research or this survey, please contact Dr Nicola Moran (nicola.moran@york.ac.uk) at the Department of Social Policy and Social Work, University of York, York, YO10 5DD. Tel: 01904 321246.

THANK YOU FOR YOUR TIME

**Consent to take part**

I understand that the information I give will be kept in strictest confidence and will only be seen by the team of researchers at the University of York. I consent to take part in the survey.

*Please tick this box if you agree to take part*

|  | 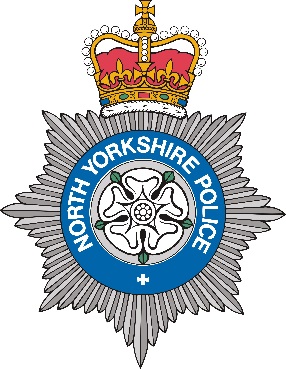 |
| --- | --- |


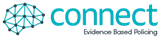

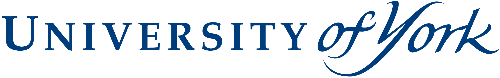


**APPENDIX 5**

**Evaluation of the ‘Connect’ mental health project**

**Information Sheet: Survey 2**

Earlier this year we asked you to complete an online survey as part of an evaluation of NYP’s involvement in the Connect project. The project included:

- A randomised controlled trial (RCT) of mental health training for front-line officers, developed in conjunction with the College of Policing; and
- Training on research methods and using research for middle and senior-ranking officers.

A significant proportion of NYP officers have been involved in at least one element of the research. It is vitally important that we identify the impact, benefit and outcomes of the training courses for NYP. To help us evaluate the impact of the training – and potentially more subtle impacts of NYP’s involvement in the Connect project – we need officers from all ranks and roles to complete this follow-up survey. Analysis of the responses you have submitted for each survey will enable the independent research team at the University of York to undertake the evaluation.

The survey has been developed using Qualtrics software that will direct you to different sets of questions depending upon your area(s) of involvement with the overall project. All police officers and police staff will be asked a small number of questions around their awareness of mental ill health, current practices within NYP around working with people in mental distress and collaborative working with partner agencies. Those who took part in the RCT will be asked an additional set of questions, while those in ranks targeted for the research methods training will be asked a different set of additional questions.

**How long will it take to complete the survey?**

Completing the survey should take no more than 10-15 minutes and will mainly involve ticking boxes. The specific questions you are asked will depend on which element(s) of the research you have been involved with.

**What are the benefits of completing the survey?**

By completing the survey you will help us to measure the effectiveness of the training and this will help us to refine and deliver training that has increased benefit to individual police officers, the force more broadly, and the individuals in mental distress who come to the attention of the police. Understanding if and how the training works will help us to refine it. This is likely to reduce demand on police resources and highlight the best way of allocating resources in the future.

**Who is undertaking the study and how is it funded?**

The Connect project is funded by the College of Policing, the Home Office, and the Higher Education Funding Council for England (HEFCE), through the Police Knowledge Fund. The research is being undertaken by an interdisciplinary team of researchers from the University of York.

**Do I have to take part in the survey?**

No, you do not have to take part in the survey. If you decide not to take part that is OK, you do not have to give a reason. If you want to take part but do not want to answer some of the questions, just leave them blank. Whether or not you decide to take part will not affect your position in NYP; nobody within NYP will be made aware of which officers have chosen not to complete the survey. However, reminders and emails encouraging officers to complete the survey will be circulated to all officers.

**Confidentiality**

All data will be anonymised, thus nobody taking part will be identifiable in the project report or any other publication. Your collar number will be removed from the survey so that all the information is anonymous. Only members of the research team will have access to the data, which will be kept on password protected computers at the University of York. Data will be securely stored for five years from the end of the study and then destroyed. All information collected during the course of the research will be kept strictly confidential in line with the Data Protection Act.

**What will happen to the information?**

Once the fieldwork is complete we will collate and analyse the data to help us determine the effectiveness of the training and of the project as a whole. This will be reported back (in anonymised form) to NYP. The research findings will also be published in report form for the funding body, and will be published in academic and policing journals.

**Ethical approval**

This evaluation of the RCT on mental health training, research methods training, and the Connect project has been approved by the Health Sciences Research Governance Committee and the Social Policy and Social Work departmental ethics committee at the University of York.

**What happens next?**

If you are willing to take the survey please click on this link [link to survey], read the section on consent and check the relevant box. Then click on ‘next’ and the survey will begin.

**Further information**

If you would like any further information on the research or this survey, please contact Dr Nicola Moran (nicola.moran@york.ac.uk) at the Department of Social Policy and Social Work, University of York, York, YO10 5DD. Tel: 01904 321246.

THANK YOU FOR YOUR TIME

**Consent to take part**

I understand that the information I give will be kept in strictest confidence and will only be seen by the team of researchers at the University of York. I consent to take part in the survey.

*Please tick this box if you agree to take part*

**Appendix 6**

**Evaluation of the ‘Connect’ mental health project**

**Evaluation Survey [TO BE DELIVERED ONLINE]**

***Tick box to confirm consenting to participate in the evaluation***

**SECTION A: Demographic data**

**(ALL)**

A1. Name of police station you report to

[drop down menu of all 39 NYP police stations for ease/speed for respondent]

A2a. Rank

[drop down menu: Volunteer, Special Constable, PCSO, Constable, Sergeant, Inspector, Police Staff (Scale 1-6), Police Staff (SO Grades), Police Staff (PO & JNC Grades), Superintendent, Chief Superintendent/Chief Inspector/Command Team]

A2b. Role

[Response, Neighbourhood, Other]

A3. No. of years working with NYP?

[Enter number]

A4. No. of years (if any) working with a different police force?

[Enter number]

A5. Age

[drop down menu of age ranges, incl. prefer not to say]

A6. Sex

[drop down menu: male, female, other]

A7. Collar number

[Enter number]

**SECTION B: Knowledge and attitudes toward mental ill health**

**(ALL)**

B1. For each of statements 1-6 below, respond by ticking one box only. Mental health problems here refer, for example, to conditions for which an individual would be seen by healthcare staff.

| **No.** | **Statement** | **Agree strongly** | **Agree slightly** | **Neither agree nor disagree** | **Disagree slightly** | **Disagree strongly** | **Don’t know** |
| --- | --- | --- | --- | --- | --- | --- | --- |
| 1 | Most people with mental health problems want to have paid employment. |  |  |  |  |  |  |
| 2 | If a friend had a mental health problem, I know what advice to give them to get professional help. |  |  |  |  |  |  |
| 3 | Medication can be an effective treatment for people with mental health problems. |  |  |  |  |  |  |
| 4 | Psychotherapy (eg talking therapy or counselling) can be an effective treatment for people with mental health problems. |  |  |  |  |  |  |
| 5 | People with severe mental health problems can fully recover. |  |  |  |  |  |  |
| 6 | Most people with mental health problems go to a healthcare professional to get help. |  |  |  |  |  |  |

B2. Say whether you think each condition is a type of mental illness by ticking one box only.

| **No.** | **Statement** | **Agree strongly** | **Agree slightly** | **Neither agree nor disagree** | **Disagree slightly** | **Disagree strongly** | **Don’t know** |
| --- | --- | --- | --- | --- | --- | --- | --- |
| 7 | Depression |  |  |  |  |  |  |
| 8 | Stress |  |  |  |  |  |  |
| 9 | Schizophrenia |  |  |  |  |  |  |
| 10 | Bipolar disorder (manic-depression) |  |  |  |  |  |  |
| 11 | Drug addiction |  |  |  |  |  |  |
| 12 | Grief |  |  |  |  |  |  |

**SECTION C: The view from the front line**

**[RCT: Only for officers taking part in the trial of mental health training for front-line officers, i.e. those in ranks PCSO, PC, Sergeant, and Inspector from Response or Neighbourhood teams from the following stations: Athena House, Fulford Road, Harrogate, Knaresborough, Malton, Northallerton, Richmond, Scarborough, Selby, Settle, Sherburn-in-Elmet, and Skipton. Automatically filtered by Qualtrics.]**

C1. Please rate your level of confidence in relation to each of the questions below by ticking one box.

| **How confident are you in…?** | **Very confident** | **Somewhat confident** | **Not very confident** | **Not at all confident** |
| --- | --- | --- | --- | --- |
| understanding the jargon/terminology associated with mental health issues? |  |  |  |  |
| recognising the signs and symptoms of a range of mental health conditions? |  |  |  |  |
| recording incidents as involving issues around mental health ? |  |  |  |  |
| responding to incidents involving people experiencing mental ill health? |  |  |  |  |
| how to refer to local specialist mental health services? |  |  |  |  |
| working with partner agencies in cases involving people with mental ill health? |  |  |  |  |
| reviewing the actions you have taken in relation to incidents involving mental ill health |  |  |  |  |

C2. Please read through the scenario below and answer the following questions.

**Scenario - part 1:**

You are called to a disturbance at a residential house. Inside the house there is a visibly distressed woman and a man who is threatening to harm the woman and himself. There has not been any violence. The man is highly agitated, he is muttering to himself, pacing up and down the living room and hitting the wall with his fist.

1. To what extent would you agree with the statement that this incident involves mental health issues? [strongly agree, slightly agree, neither agree nor disagree, slightly disagree, strongly disagree]
2. If you were the first responder, what would you do? [free text]
3. what would be your three most important considerations/priorities? [free text]

**Scenario – part 2:**

You feel that, in the circumstances, you cannot make an arrest or take any further action; however, you feel uncomfortable to simply leave the couple.

1. (i) Which agency or service would you consider making a referral to and why? [free text]
2. How confident would you feel in making a referral? [very confident, somewhat confident, neither confident nor unconfident, a little unconfident, very unconfident]
3. How confident would you feel in working with other agencies (please specify which)? [very confident, somewhat confident, neither confident nor unconfident, a little unconfident, very unconfident]
4. how confident do you feel in the actions you have taken? [very confident, somewhat confident, neither confident nor unconfident, a little unconfident, very unconfident]
5. Any other comments in relation to this scenario? [free text]

C3. How do you think relationships between NYP and local mental health services could be improved? [free text]

C4. Communicating with people. Please read each statement below and then click on the number in the right hand column indicating the extent to which you agree with the statement.

| **Statement** | ***Level of agreement*** |
| --- | --- |
| I find it difficult to build a rapport with people experiencing mental ill health | *Strongly agree 1 2 3 4 5 6 7 Strongly disagree* |
| I can empathise with the people with mental ill health I have to deal with on a regular basis | *Strongly agree 1 2 3 4 5 6 7 Strongly disagree* |
| I can usually predict how someone experiencing mental ill health will behave before speaking to them | *Strongly agree 1 2 3 4 5 6 7 Strongly disagree* |
| I can develop good relationships with the people with mental ill health I have regular  contact with | *Strongly agree 1 2 3 4 5 6 7 Strongly disagree* |
| I treat people with mental ill health with respect regardless of how they treat me | *Strongly agree 1 2 3 4 5 6 7 Strongly disagree* |
| If a member of the public is rude to me, I will be less polite to them | *Strongly agree 1 2 3 4 5 6 7 Strongly disagree* |
| I don’t lose my patience when dealing with difficult members of the public | *Strongly agree 1 2 3 4 5 6 7 Strongly disagree* |
| I find it difficult to reassure members of the public who are upset | *Strongly agree 1 2 3 4 5 6 7 Strongly disagree* |
| I show my irritation to members of the public who repeatedly don’t do what I ask | *Strongly agree 1 2 3 4 5 6 7 Strongly disagree* |
| I find it easy talking to people who I think are anti-police | *Strongly agree 1 2 3 4 5 6 7 Strongly disagree* |
| I remain calm when dealing with people who are agitated | *Strongly agree 1 2 3 4 5 6 7 Strongly disagree* |
| If there is nothing more I can do for a person experiencing mental ill health, I put them in touch with someone who can help | *Strongly agree 1 2 3 4 5 6 7 Strongly disagree* |
| My previous training has given me the skills necessary to talk to people experiencing mental ill health | *Strongly agree 1 2 3 4 5 6 7 Strongly disagree* |
| When a person is speaking, I intentionally make eye contact with them to show I am paying attention | *Strongly agree 1 2 3 4 5 6 7 Strongly disagree* |
| My previous training has taught me about the kind of service people with mental ill health want from us | *Strongly agree 1 2 3 4 5 6 7 Strongly disagree* |
| I try to a mirror a person’s body language to build a good rapport with them | *Strongly agree 1 2 3 4 5 6 7 Strongly disagree* |
| My previous training has given me the chance to think about how I speak to people with mental ill health | *Strongly agree 1 2 3 4 5 6 7 Strongly disagree* |
| My previous training has given me confidence to deal with members of the public | *Strongly agree 1 2 3 4 5 6 7 Strongly disagree* |
| The way I interact with people has improved as a result of my previous training | *Strongly agree 1 2 3 4 5 6 7 Strongly disagree* |
| My previous training has prepared me to deal with difficult interactions with the public | *Strongly agree 1 2 3 4 5 6 7 Strongly disagree* |
| To help build rapport with people, I tell them who I am | *Strongly agree 1 2 3 4 5 6 7 Strongly disagree* |
| I’m not comfortable giving people my first name | *Strongly agree 1 2 3 4 5 6 7 Strongly disagree* |
| I deliberately nod when a person is talking to let them know I am listening | *Strongly agree 1 2 3 4 5 6 7 Strongly disagree* |

C5. To what extent do you feel that the training you have had around working with people with mental ill health has been sufficient? [strongly agree, somewhat agree, neither agree nor disagree, somewhat disagree, strongly disagree]

C6. In which areas, if any, do you feel you lack confidence when dealing with a person with mental ill health? [free text]

*Post survey only: C7. To what extent, if any, do you feel that the training [the station’s involvement in the trial of the mental health training] impacted on:*

1. *the work of the station? [a lot, a little, not at all] [free text: please elaborate, explain, provide an example]*
2. *your own work? [a lot, a little, not at all] [free text: please elaborate, explain, provide an example]*
3. *people with mental health problems? [a lot, a little, not at all] [free text: please elaborate, explain, provide an example]*

*Post survey only: C8. We are seeking to interview a small number of officers who received the mental health training delivered by mental health practitioners between May and July 2016. The interview would involve discussing in more detail your experiences of the training programme and any impact this has had on your day to day work. If you would like to find out more about taking part in a confidential interview, please tick the box below.*

*Yes, I would like to find out more about taking part in a follow-up interview:*

**SECTION D: Current practices within NYP**

**(All)**

D1. Regarding interactions with members of the public who are believed to be suffering from mental ill health, please indicate your thoughts on the effectiveness of current practices within NYP:

|  |  | Very good | Satisfactory | Less than satisfactory (some improvement needed) | Poor (much improvement needed) |
| --- | --- | --- | --- | --- | --- |
| a | Identification of mental vulnerability |  |  |  |  |
| b | Recording of relevant information |  |  |  |  |
| c | Responding in a manner that best meets the needs of the individual in mental distress |  |  |  |  |
| d | Referring to appropriate services |  |  |  |  |
| e | Reviewing incidents for effective risk management |  |  |  |  |

f) If you believe improvements are needed in any area outlined in a-e above, do you have suggestions how practice could be improved? [free text]

D2a. In relation to working with partners (e.g. NHS, College of Policing, Universities) to improve practices around working with people experiencing mental ill health (with respect to identification, recording, responding, referring, and reviewing) do you currently think collaborative working is:

[very good, satisfactory, less than satisfactory (some improvement needed), poor (much improvement needed)]?

D2b. If you think collaborative working could be improved, with which specific partner? In which area? And how? [free text]

**SECTION E: Understanding and use of research**

**(Only for officers in the ranks eligible for the research methods training – Inspector and above. This set of questions will not be asked of front-line PCSOs, Constables and Sergeants who attended the mental health training. A small number of Inspectors who attended the mental health training will also be asked to complete this question set)**

E1. Please name a specific approach that you have used in the last six months to improve your professional practice. This might be a new method, product or initiative.

E2. Which, if any, of the following were important in identifying the approach you named above?

Please select the three most important options:

- Ideas generated by me or my station
- Ideas from officers elsewhere
- Advice from senior officers/NYP
- Articles, reports, books or summaries based on academic research
- Articles, reports, books or summaries based on police officer experience
- Promotional materials from an external supplier
- Research conducted by me or my colleagues
- Information from training or CPD
- Guidance from ACPO/College of Policing
- Don’t know
- Other (Specify)

E3. What does the term “evidence-based policing” mean to you?

Please select up to three options that best describe your understanding of the term:

- Learning from colleagues and applying that learning
- Applying ACPO/College of Policing guidance
- Using an online evidence database
- Combining academic research evidence with my professional expertise
- Using intelligence to make decisions about priorities and tactical options
- Applying the recommendations of an external supplier
- Reading and applying information from academic research or from working with researchers
- Learning from external consultants. trainers or advisors
- I don’t know

E4. This question aims to find out how, if at all, you use research information in your work. By ‘research’ we mean information from books, reports, articles, summaries, training or events that is based on academic studies.

Please indicate the extent to which you agree or disagree with the following:

(Strongly agree/agree/neither agree nor disagree/disagree/strongly disagree)

- Information from research plays an important role in informing my policing practice
- I do not believe that using information from research will help to improve policing outcomes
- I know where to find relevant research that may help to inform my methods/practice
- My superior officers do not encourage me to use information from research to improve my practice
- I am able to relate information from research to my context
- Other officers I know rarely use information to inform their practice
- I feel confident about analysing information from research
- Information from research conducted elsewhere is of limited value to me
- I use information from research to help me decide how to implement new approaches

E5. Listed below are three statements of research purpose (descriptions of why somebody might want to carry out research). Click on the drop down menu next to each research purpose and select what you think is most appropriate research method for achieving that purpose. You can only select one research method for each research purpose.

Statements of research purpose:

1. To provide an overview of the evidence base;
2. To determine whether an intervention or approach has a direct impact on policing outcomes;
3. To understand how an intervention or approach works in practice.

Research methods:

1. Randomised controlled trial;
2. Longitudinal study;
3. Interviews and/or questionnaires;
4. Literature review;
5. Correlational study.

*Post only*

*E6a. Did you attend the research methods training course (Sept/Oct 2016) run by academics from the University of York? [yes/no/comment]*

*E6b. If yes, to what extent do you think the training increased your:*

- *appreciation of the value of using research to inform practice? [a lot, a little, not at all]*
- *understanding of different research methods? [a lot, a little, not at all]*
- *Use of research/evidence to inform practice? [a lot, a little, not at all]*
- *Please provide an example [free text]*

*E6c. How satisfied were you with the following elements of the training:*

- *The content of the training? [very satisfied, quite satisfied, neither satisfied nor dissatisfied, a little unsatisfied, very unsatisfied]*
- *The applicability of the training to your work? [very satisfied, quite satisfied, neither satisfied nor dissatisfied, a little unsatisfied, very unsatisfied]*

**APPENDIX 7**

**Deputy Chief Superintendent trailer (email)**

**EMAIL HEADER: Forthcoming survey on mental health**

In the next few days, you will receive an invitation to participate in a very important online survey on mental health. I strongly encourage you to take part, and give researchers the benefit of your valuable insights and experiences. The study’s success depends on as many people responding as possible.

The survey forms part of an **independent** **evaluation of a new mental health training programme for front-line officers, use of research/research methods training for middle-senior ranking officers, and an evaluation of the overall programme of training and research.** Officers will be presented with different sets of questions depending on their station and rank. The survey is a priority for our force, and will be very important in shaping future training in England and Wales as the findings will feed into College of Policing training. The survey will be conducted and analysed by researchers at the **University of York**. It should take about **15 minutes to complete.** The survey is due to close on **[date]**.

**All survey answers will be confidential. Individual responses will be shared only among a select group of researchers working at the University of York, and will NOT be shared with force managers or colleagues. Research results will be presented in ways that do not allow individual participants to be identified.**

Please look out for your invitation to participate. If you have any queries, do not hesitate to contact Inspector Bill Scott or Dr Nicola Moran at the University of York (nicola.moran@york.ac.uk).

Sincerely,

Deputy Chief Constable Tim Madgwick

**APPENDIX 8**

**Deputy Chief Superintendent follow-up (email)**

**EMAIL HEADER: Final request for your participation in mental health survey**

I am writing, one last time, to remind you to complete the online survey on mental health. If you have already started the survey, I would be grateful if you could find the time to complete the remaining questions.

Please also remember, **it does not matter whether you are scheduled to attend one of the new training sessions or not – everyone I write to is being asked to participate.**

The deadline for you to complete this **independent** survey has been extended to **[date]**.

The response has been good so far, but I would like **all officers** to take part because mental health is an important and high profile issue. The survey forms part of an **independent** **evaluation of a new mental health training programme for front-line officers, use of research/research methods training for middle-senior ranking officers, and an evaluation of the overall programme of training and research.** The survey is being conducted by researchers at the **University of York** and is an independent evaluation.

Your answers to these questions are very important to the overall success of the survey. The results will help shape future training on mental health and evidence-based policing. I hope you are able to give the researchers the benefit of your insights and experiences.

I can reassure you that your answers will be kept **confidential** and that you will not be identified when the University of York reports its findings. **No-one** in the force will have access to your individual responses. Your answers will be stored securely at the University, and only a select group of researchers working at the University will have access to them.

You will receive another e-mail with a survey link in the next day. However, the link you received in earlier e-mails remains active and you can use this now.

If you have any queries, do not hesitate to contact Inspector Bill Scott or Dr Nicola Moran at the University of York ([nicola.moran@york.ac.uk](mailto:nicola.moran@york.ac.uk)).

Yours sincerely,

Deputy Chief Constable Tim Madgwick

|  | 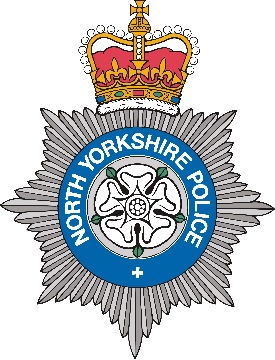 |
| --- | --- |

**
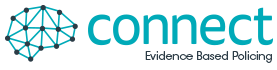
**
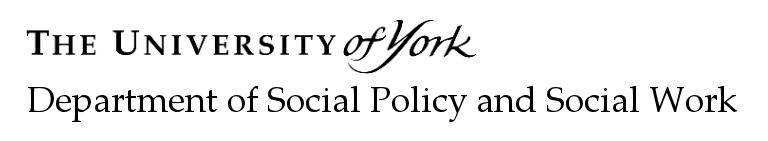


**APPENDIX 9**

**Evaluation of the Mental Health Training for Front Line Police Officers: Follow-up interviews**

**Information Sheet**

Police officers from across a number of police stations within North Yorkshire Police (NYP) have been taking part in a randomised controlled trial of mental health training for front-line police officers. We are contacting you personally at this stage of the study as you indicated in the recent evaluation survey that you would like to find out more about taking part in a follow-up interview. Thank you for your willingness to find out more about this and for completing the evaluation surveys.

**What is the research about and what will be asked in the interview?**

We want to find out more about the extent of any impact of the mental health training on officers’ knowledge of, and attitudes toward, working with people experiencing mental ill health, and on the day to day work of officers, the work of the station and on people experiencing mental ill health. In this final stage of the research we aim to interview about 20 front-line officers who received the mental health training. If you agree to participate, we would like to discuss with you, in more detail than was possible in the survey, your experience of the training intervention and any impact you feel this has had. The interview is likely to take around 30 minutes, depending on how much time you have available and how much you tell us.

**Who is undertaking the study and how is it funded?**

This element of the study is being undertaken by researchers from the Department of Social Policy and Social Work at the University of York: Professor Martin Webber and Dr Nicola Moran. The project is funded by the College of Policing, the Home Office, and the Higher Education Funding Council for England (HEFCE), through the Police Knowledge Fund.

**Do I have to take part in an interview?**

No, you do not have to take part in an interview. If you decide not to take part that is OK, you do not have to give a reason. If you want to take part but do not want to answer some of the questions, that is also fine. You are also free to withdraw from the interview at any point, without needing to give a reason. Whether or not you decide to take part will not affect your position in NYP; nobody within NYP will be made aware of which officers have or have not chosen to take part in an interview.

**Informed consent**

If you do agree to take part, the researcher will go through the information sheet with you and answer any questions you may have about the research prior to the interview. If you remain willing to take part in an interview, the researcher will ask you to complete and sign a consent form prior to the interview commencing. We would like to audio record the interview and have it professionally transcribed to help us with the analysis. If you prefer not to be audio recorded, the researcher will instead take detailed hand-written notes. Please be assured that everything you tell us would be in confidence (see below).

**Confidentiality**

All information which is collected during the course of the research will be kept strictly confidential in line with the Data Protection Act. The transcription of the interview will be anonymised (your name, role and any other identifying comments will be removed) so that nobody taking part in the project will be identifiable in the project report or any other publication. The anonymised transcription will only be accessible to members of the research team working on this element of the study. It will be stored on a secure password-protected server at the University of York for five years from the end of the study and then destroyed.

**What will happen to the information?**

Once the trial is over we will collate and analyse the data to determine the impact and outcomes of the training intervention. This will be reported back to NYP. The results of the trial will also be published in report form for the funding body (the Home Office, the College of Policing, and HEFCE), and will be published in academic and policing journals. The report will include recommendations about future mental health training for police officers.

**Disclosure of harm**

If, in the unlikely event that you disclose something during the interview which suggests that you or somebody else is at risk of harm, the researcher will have to share this information with others who may be required to act.

**Ethical approval**

This evaluation of the mental health training has been approved by the Department of Health Sciences Research Governance Committee at the University of York.

**What happens next?**

If you would like to take part in an interview please contact Nicola by email at [nicola.moran@york.ac.uk](mailto:nicola.moran@york.ac.uk) or telephone: 01904 321246 to arrange a time, date and location for the interview.

**Further information**

If you would like any further information about the research, please contact Dr Nicola Moran (nicola.moran@york.ac.uk) at the Department of Social Policy and Social Work, University of York, York, YO10 5DD. Tel: 01904 321246.

THANK YOU FOR YOUR TIME

|  | 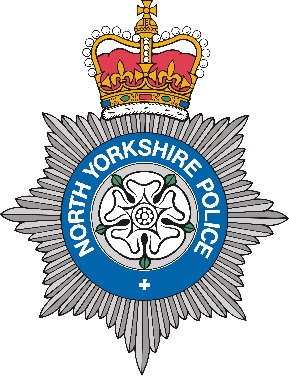 |
| --- | --- |

**
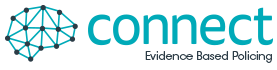
**
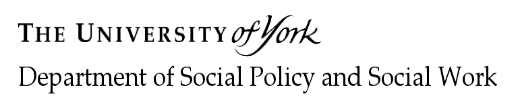


**APPENDIX 10**

**Evaluation of the Mental Health Training for Front Line Police Officers: interviews**

**Consent Form**

**Please tick:**

| I have read the information sheet and understand the purpose of the study and what is involved | - Yes | - No |
| --- | --- | --- |
| I understand that the study is strictly confidential and I will not be named in any study reports. | - Yes | - No |
| I understand that participation is voluntary and that I can withdraw from the study at any time without giving a reason and this will not affect my position. | - Yes | - No |
| I understand that if I disclose something during the interview which suggests that I or somebody else is at risk of harm, the researcher will have to share this information with others who may be required to act. | - Yes | - No |
| I am willing to be interviewed as part of this research. | - Yes | - No |
| I agree for my interview to be digitally recorded. | - Yes | - No |

**Participant name: …...………………………………………………………**

**Collar No.: …………………………………………………………………….**

**Signature: …………………………………………………………………….**

**Date: ………………………………………….......**

**Name of researcher: ………………………………………………………..**

**Signature: ………………..……………………………………………………**

**Date: ………………………………………………**

|  |
| --- |
|  |
|  |
|  |
|  |
|  |

**APPENDIX 11
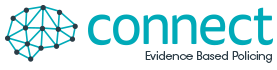
**

**Mental health awareness training for front-line police officers**

**Support for officers**

Thank you very much for taking part in this training. With one in six of the population experiencing mental health problems at any one time it is possible that the training has raised some issues or concerns or stirred up memories or experiences in relation to yourself, family, friends or colleagues. If you would like further information or advice, or if you would like to talk about your experiences and feelings, you may wish to contact your local GP or one of the following organisations:

**Blue Light Infoline**

The Blue Light Infoline offers confidential, independent and practical support, advice and signposting around mental health and wellbeing. The Infoline is just for emergency service staff, volunteers and their families, to help keep you or those you care about well for work. Open Monday to Friday, 9am to 6pm, the phone number is charged at local rates. You can also contact Blue Light at any time using the email or text details, for a response from one of the dedicated advisors during the Infoline working hours.

The team provides information on a range of topics including:

- staying mentally healthy for work
- types of mental health problems
- how and where to get help
- medication and alternative treatments
- advocacy
- Post Traumatic Stress Disorder (PTSD)
- existing emergency service support
- mental health and the law.

Blue Light will look for details of help and support in your own area or that is relevant to your emergency service. Unfortunately advisors are unable to offer an emotional listening service or counselling through the Infoline.

**Telephone:** 0300 303 5999 (09:00-18:00 Mon-Fri)

**Email:** bluelightinfo@mind.org.uk

**Text:** 84999

**Website:** [www.mind.org.uk/news-campaigns/campaigns/bluelight/blue-light-infoline/](http://www.mind.org.uk/news-campaigns/campaigns/bluelight/blue-light-infoline/)

**SaneLine**

SANE runs a national, out-of-hours mental health helpline offering specialist emotional support and information to anyone affected by mental illness, including family, friends and carers. Open every day of the year from 6pm to 11pm.

**Telephone:** 0300 304 7000 (18:00-23:00 daily)

**Website:** [www.sane.org.uk/what_we_do/support/helpline/](http://www.sane.org.uk/what_we_do/support/helpline/)

**Samaritans**

The Samaritans is a national organisation providing information and support to individuals with a range of concerns. If there's something troubling you, then get in touch. We're here 24 hours a day, 365 days a year. If you need a response immediately, it's best to call us on the phone. This number is FREE to call from a landline or mobile.

**Telephone:** 116 123

**Email:** [jo@samaritans.org](mailto:jo@samaritans.org)

**Website:** [www.samaritans.org/how-we-can-help-you/contact-us](http://www.samaritans.org/how-we-can-help-you/contact-us)

**North Yorkshire out-of-hours mental health helpline**

A confidential and anonymous service available to anyone registered with a North Yorkshire GP. Providing a listening ear, emotional support, signposting and referral to other services for individuals and the carers of people experiencing mental distress.

**Telephone:** 0333 0000 309 (Mon-Thurs 17:00-08:30, open Friday from 16:30, open 24 hours at the weekends including bank holidays)

**Website:** [www.northyorks.gov.uk/article/24057/What-can-I-do-in-a-crisis-or-an-emergency](http://www.northyorks.gov.uk/article/24057/What-can-I-do-in-a-crisis-or-an-emergency)

|  |  |
| --- | --- |


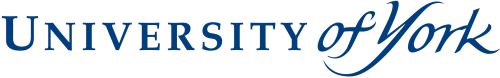


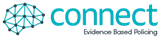


**Appendix 12**

**Post-training feedback form**

As part of the evaluation of the mental health training for front-line officers, we would like to know what you thought of the course you just attended. Please read the following statements and circle the number that best describes the extent to which you agree or disagree with each one (where 1 is ‘Strongly agree’ and 7 is ‘Strongly disagree’).

1. Overall, I was satisfied with the training course I attended

*Strongly agree 1 2 3 4 5 6 7 Strongly disagree*

2. I developed practical skills on the course that will help me improve the contact I have with people experiencing mental ill health

*Strongly agree 1 2 3 4 5 6 7 Strongly disagree*

3. I received helpful feedback during the course

*Strongly agree 1 2 3 4 5 6 7 Strongly disagree*

4. The course material was covered too quickly

*Strongly agree 1 2 3 4 5 6 7 Strongly disagree*

5. I learnt something new from the training

*Strongly agree 1 2 3 4 5 6 7 Strongly disagree*

6. The activities gave me the chance to practice what I had learnt

*Strongly agree 1 2 3 4 5 6 7 Strongly disagree*

7. I was not given enough time to think about how I would apply the training in my work

*Strongly agree 1 2 3 4 5 6 7 Strongly disagree*

8. I thought the scenarios were realistic

*Strongly agree 1 2 3 4 5 6 7 Strongly disagree*

9. I have more questions than answers following the training

*Strongly agree 1 2 3 4 5 6 7 Strongly disagree*

10. I can see how the training will help me in my day-to-day work

*Strongly agree 1 2 3 4 5 6 7 Strongly disagree*

Any further comments: ………………………………………………………………………………………………………………… …………………………………………………………………………………………………………………………………………………………………………………………………………………………………………………………………………………………………………………………………………………………………………………………………………………………………………………………………………………………………………………………………………………………………………………………………………………………………………

**Thank you for your feedback**

**Appendix 13: Flow diagram for the Connect Trial**

*
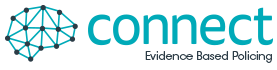
*

Intervention n=6

Control n=6

Routinely collected trial data

Online Survey

Routinely collected trial data

Online Survey

Online Survey

Qualitative interviews

Online Survey

Eligible Police Stations n=12

Randomisation of police stations

Specialised mental health training package

Routine Training

**Baseline measures**

**Intervention**

**Post-intervention measures**
