# Supplementary material for: Effectiveness of a training program for police officers who come into contact with people with mental health problems: A pragmatic randomised controlled trial
Source: PLoS One. 2017 Sep 8;12(9):e0184377. doi: 10.1371/journal.pone.0184377 (PMC5590916; doi:10.1371/journal.pone.0184377)
Supplement: S1 Table — (DOCX) [file pone.0184377.s003.docx]

| **TIDIER CHECKLIST ITEM** | **Intervention details** |
| --- | --- |
| *Item* *1*. Brief name: provide the name or a phrase that describes the intervention | A bespoke mental health training package for frontline police officers |
| *Item 2.* Why: Describe any rationale, theory, or goal of the elements essential to the intervention | Mental health training for frontline police officers may help them to: identify mental vulnerability; record relevant information using available systems; respond using appropriate internal and external resources; refer vulnerable people into services to provide longer-term assistance and review incidents to make sure that risks have been effectively managed The training aimed to reduce demand on police resources through improving officers’ knowledge, awareness and understanding of mental ill health and vulnerability, referral pathways and the skills necessary to work both with people in mental health distress and with partner agencies  The content of the training was informed by College of Learning Standards for mental health training for police officers and a systematic review of mental health training for non-mental health trained professionals. |
| *Item 3:* What (materials): Describe any physical or informational materials used in the intervention, including those provided to participants or used in intervention delivery or in training of intervention providers. Provide information on where the materials can be accessed.  *Item 4.* What (Procedures) Describe each of the procedures, activities and/or processes used in the intervention, including any enabling or support activities. | The intervention group received a bespoke mental health training package. The training package involved traditional classroom style delivery of information; skills based learning on how to communicate with people in mental health crisis; working through scenarios in small groups to look at different approaches to engaging and communicating with people in mental distress and to provide opportunities for officers to discuss real world scenarios and seek advice; exploration of the types of mental health support services and organisations that exist locally; talking head video clips of individuals from various partner agencies and discussion and skills practice around the importance of reviewing actions taken to ensure the best interests of the person in mental health distress. For further details of the development and delivery of the intervention please see [[22](#_ENREF_22)] |
| *Item 5:* Who provided: For each category of intervention provider describe their expertise, background and any specific training given. | Qualified mental health professionals from a local NHS mental health trust delivered the training. |
| *Item 6:* How: describe the modes of delivery of the intervention and whether it was provided individually or in a group. | The intervention was face-to-face by mental health professionals to groups of frontline officers. The intervention was delivered using: lecture style delivery; small group discussion; filmed scenarios; short films with experts that had experience of living with a mental health condition and contact with NYP during a mental health crisis and talking head videos with 11 mental health services and partner agencies. |
| *Item 7:* Where: describe the type of location where the interventions occurred, including any necessary infrastructure or relevant features | The intervention was delivered at 3 police locations in North Yorkshire. |
| *Item 8:* When and how much: Describe the number of times the intervention was delivered and over what period of time including the number of sessions; their schedule and their intensity or dose. | The intervention was a one-day mental health training package delivered across 25 training days between May and August 2016. |
| *Item 9.* Tailoring: If the intervention was planned to be personalised, titrated or adapted, then describe what, why, when and how. | Exploration of the types of mental health support services and organisations that exist locally were tailored to each local area. |
| *Item 10:* Modifications: if the intervention was modified during the course of the study, describe any changes (what, when, why and how). | None |
| *Item 11:* How well (planned): if intervention adherence or fidelity was assessed describe how and by whom, and if any strategies were used to maintain or improve fidelity, describe them. | The training was co-produced by the research team, NYP and mental health professionals. All mental health professionals who delivered the training were briefed by a member of the research team in the days and weeks leading up to delivery of the training. Mental health professionals were also provided with a lesson plan to guide them through the training day and various activities. |
| *Item 12:* How well (actual): if intervention adherence or fidelity was assessed, describe the extent to which the intervention was delivered as planned. | The total number of officers put forward for training was 360. Of these, 249 officers received the specialised mental health training intervention; 224 from stations allocated to the intervention group, 15 from stations allocated to the control group, and 10 from non-trial stations. Reasons for non-attendance included: training not required/appropriate; officer on maternity adjustment; restricted duties; sickness and other/unknown. Nineteen attendees were trained inappropriately as they were not of a rank eligible for the intervention. |
